# Non-invasive fluorescence sensing reveals changes in intestinal barrier function and gastric emptying rate in a first-in-human study of Crohn’s disease

**DOI:** 10.1177/17562848251361634

**Published:** 2025-08-13

**Authors:** Jonathan Gan, Qian Chen, Elena Monfort Sanchez, Nilanjan Mandal, Jiacheng Xu, Zixin Wang, Arjun Agarwal, Emmanuel Oluwatunmise, Pratik Ramkumar, Ash Salam, Elena Chekmeneva, María Gómez-Romero, Lynn Maslen, Sharmili Balarajah, Robert Perry, Karl King Yong, Jonathan Hoare, Nick Powell, James Alexander, James Avery, Hutan Ashrafian, Ara Darzi, Alex J. Thompson

**Affiliations:** The Hamlyn Centre, Institute of Global Health Innovation (IGHI), Imperial College London, London, UK; Department of Surgery & Cancer, St Mary’s Hospital, Imperial College London, London, UK; The Hamlyn Centre, Institute of Global Health Innovation (IGHI), Imperial College London, London, UK; Department of Surgery & Cancer, St Mary’s Hospital, Imperial College London, London, UK; The Hamlyn Centre, Institute of Global Health Innovation (IGHI), Imperial College London, London, UK; Department of Surgery & Cancer, St Mary’s Hospital, Imperial College London, London, UK; The Hamlyn Centre, Institute of Global Health Innovation (IGHI), Imperial College London, London, UK; Department of Surgery & Cancer, St Mary’s Hospital, Imperial College London, London, UK; The Hamlyn Centre, Institute of Global Health Innovation (IGHI), Imperial College London, London, UK; Department of Surgery & Cancer, St Mary’s Hospital, Imperial College London, London, UK; The Hamlyn Centre, Institute of Global Health Innovation (IGHI), Imperial College London, London, UK; Department of Surgery & Cancer, St Mary’s Hospital, Imperial College London, London, UK; The Hamlyn Centre, Institute of Global Health Innovation (IGHI), Imperial College London, London, UK; Department of Surgery & Cancer, St Mary’s Hospital, Imperial College London, London, UK; The Hamlyn Centre, Institute of Global Health Innovation (IGHI), Imperial College London, London, UK; Department of Surgery & Cancer, St Mary’s Hospital, Imperial College London, London, UK; The Hamlyn Centre, Institute of Global Health Innovation (IGHI), Imperial College London, London, UK; Department of Surgery & Cancer, St Mary’s Hospital, Imperial College London, London, UK; National Phenome Centre, Section of Bioanalytical Chemistry, Department of Metabolism, Digestion and Reproduction, Imperial College London, London, UK; National Phenome Centre, Section of Bioanalytical Chemistry, Department of Metabolism, Digestion and Reproduction, Imperial College London, London, UK; National Phenome Centre, Section of Bioanalytical Chemistry, Department of Metabolism, Digestion and Reproduction, Imperial College London, London, UK; National Phenome Centre, Section of Bioanalytical Chemistry, Department of Metabolism, Digestion and Reproduction, Imperial College London, London, UK; Department of Metabolism, Digestion and Reproduction, Imperial College London, London, UK; Gastroenterology Department, Imperial College Healthcare NHS Trust, St Mary’s Hospital, London, UK; Department of Metabolism, Digestion and Reproduction, Imperial College London, London, UK; Gastroenterology Department, Imperial College Healthcare NHS Trust, St Mary’s Hospital, London, UK; Gastroenterology Department, Imperial College Healthcare NHS Trust, St Mary’s Hospital, London, UK; Department of Surgery & Cancer, St Mary’s Hospital, Imperial College London, London, UK; Gastroenterology Department, Imperial College Healthcare NHS Trust, St Mary’s Hospital, London, UK; Department of Metabolism, Digestion and Reproduction, Imperial College London, London, UK; Gastroenterology Department, Imperial College Healthcare NHS Trust, St Mary’s Hospital, London, UK; Department of Metabolism, Digestion and Reproduction, Imperial College London, London, UK; Gastroenterology Department, Imperial College Healthcare NHS Trust, St Mary’s Hospital, London, UK; IBD Unit, St Mark’s National Bowel Hospital, Acton Ln, London, UK; The Hamlyn Centre, Institute of Global Health Innovation (IGHI), Imperial College London, London, UK; Department of Surgery & Cancer, St Mary’s Hospital, Imperial College London, London, UK; The Hamlyn Centre, Institute of Global Health Innovation (IGHI), Imperial College London, London, UK; Department of Surgery & Cancer, St Mary’s Hospital, Imperial College London, London, UK; The Hamlyn Centre, Institute of Global Health Innovation (IGHI), Imperial College London, London, UK; Department of Surgery & Cancer, St Mary’s Hospital, Imperial College London, London, UK; The Hamlyn Centre, Institute of Global Health Innovation (IGHI), Imperial College London, Exhibition Road, South Kensington, London SW7 2AZ, UK; Department of Surgery & Cancer, St Mary’s Hospital, Imperial College London, South Wharf Road, South Kensington, London W2 1NY, UK

**Keywords:** Crohn’s disease, fluorescence, gastric emptying, gut barrier function, inflammatory bowel disease, non-invasive, sensing

## Abstract

**Background:**

Crohn’s disease is characterised by multifaceted changes in gut function, involving not just inflammatory effects but also alterations in gut barrier function and gastric motility. However, current diagnostic tools used to measure key gut functional parameters are invasive, unreliable or time-consuming. Thus, we applied a novel, non-invasive fluorescence sensing technology – transcutaneous fluorescence spectroscopy (TFS) – to investigate gut barrier function and gastric emptying in Crohn’s disease.

**Objectives:**

Our study aimed to validate TFS for non-invasive gastrointestinal (GI) diagnostics and to explore changes in gut barrier function and gastric emptying rate simultaneously in Crohn’s disease.

**Design:**

A cross-sectional study involving patients with Crohn’s disease and healthy individuals.

**Methods:**

We performed fluorescent measurements and lactulose:mannitol (L:M) tests in 38 Crohn’s disease patients and 20 healthy volunteers. We investigated multiple TFS-derived parameters as indicators of gut barrier function and gastric emptying rate. Using these parameters, we assessed differences between healthy volunteers, inactive Crohn’s patients and active Crohn’s patients, and calculated correlations between TFS and L:M values.

**Results:**

TFS-derived parameters revealed significantly increased intestinal permeability and delayed gastric emptying in patients with active Crohn’s compared to healthy controls. TFS trends showed encouraging alignment with those from the L:M test, suggesting potential concordance with established methods. No adverse events were reported.

**Conclusion:**

TFS enables rapid, non-invasive discrimination of Crohn’s patients from healthy volunteers and allows simultaneous assessment of gut barrier function and gastric emptying rate – two important aspects of GI function in Crohn’s disease. This implies potential for improved monitoring and diagnosis of Crohn’s disease (and other gut disorders) as well as more advanced study of gut function in health and disease.

**Trial registration:**

The clinical study reported in this article was registered with ClinicalTrials.gov prior to enrolment of the first participant. https://clinicaltrials.gov/study/NCT03434639 and Registration number: NCT03434639.

## Plain language summary

Using light for rapid, non-invasive assessment of gut health

The function of the gut is altered in a wide range of clinical conditions, from inflammatory bowel disease (IBD) to undernutrition. However, clinical tests used to measure gut function are not widely used, as they are typically expensive, slow and uncomfortable for the patient.

We developed a new light-based technology for rapid assessment of gut health. Using this approach – known as transcutaneous fluorescence spectroscopy (TFS) – patients first consume a drink containing a fluorescent dye. A wearable probe is then attached to the finger to detect the uptake of that dye from the gut into the blood stream in a completely non-invasive manner (by shining light through the skin). By analysing the resulting signals, it is then possible to assess numerous aspects of gut health.

We applied TFS to a study of Crohn’s disease (a form of IBD) to investigate changes in the gut barrier (which separates the internal contents of the gut from the rest of the body) and the rate of stomach emptying. These two key elements of gut function are known to be impaired in IBD but are challenging to measure simultaneously. Using TFS, we assessed gut barrier integrity and stomach emptying simultaneously, and showed that both were significantly different in Crohn’s disease patients than in healthy volunteers.

Overall, this suggests that TFS has potential for improved monitoring and diagnosis of gut disorders as well as more advanced study of gut function in health and disease.

## Introduction

Crohn’s disease is a subset of inflammatory bowel disease (IBD) that can affect any part of the gastrointestinal tract. It has a chronic, unpredictable course that can severely impact a patient’s quality of life, and its incidence is increasing worldwide.^1^ The pathogenesis of Crohn’s disease is multifactorial, with genetic, immunological and environmental factors all associated with onset and progression.^2^

While characterised by chronic inflammation in the gut, other changes in gastrointestinal (GI) function are also observed in Crohn’s disease (and in IBD in general), including impaired gut barrier function^3–8^ and reduced gastric motility.^9–14^ Indeed, numerous studies have highlighted the importance of impaired gut barrier function (or increased intestinal permeability) in the pathophysiology of IBD (e.g. Meddings^4^ and Prasad et al.^5^), suggesting that increased permeability may play a causative (rather than simply correlative) role in IBD.^7,8^ Nonetheless, the role of gut barrier function (and gastric motility) in the pathogenesis of Crohn’s disease is still not fully understood.

Despite the importance of gut barrier function in disease, the tools and techniques currently used to assess intestinal permeability are either highly invasive (e.g. endoscopic biopsy), unreliable and/or difficult to perform in certain groups (e.g. infants, patients with reduced urinary output, etc.).^6,15,16^ Dual sugar assays such as the lactulose:mannitol (L:M) and lactulose:rhamnose (L:R) tests have been widely used to assess gut barrier function (including in Crohn’s disease^17–21^). However, these assays suffer from numerous limitations, including unreliability in patients where longitudinal urine collection is challenging, delays in reporting of results and high heterogeneity of data.^6^

To address these limitations, we have developed a novel fluorescent technology for rapid, non-invasive assessment of intestinal barrier function using fluorescein (a clinically approved fluorescent contrast agent).^22–26^ This technique – transcutaneous fluorescence spectroscopy (TFS) – allows for real-time, non-invasive assessment of the translocation of orally ingested fluorescent contrast agents from the gut into the bloodstream.^22–25,27,28^ By appropriately analysing the fluorescence dynamics, it is possible to obtain readouts of both intestinal permeability^24^ and gastric emptying rate.^23^ Crucially, TFS is almost completely non-invasive and does not require the collection of biological samples such as blood or urine. Furthermore, data can be analysed immediately upon collection, as the transcutaneous signal negates the need for laboratory-based analysis of collected samples.

In this study, we applied TFS to a clinical investigation of gut function in Crohn’s disease patients and healthy volunteers. Our results demonstrated statistically significant differences between Crohn’s patients and healthy volunteers in terms of both intestinal permeability and gastric emptying rate (two parameters that are rarely measured in combination and which are often considered confounders – for example, delayed gastric emptying can confound measurements of permeability when using L:M and L:R tests). This highlights the potential of TFS to improve the assessment of gut function in health and disease and to enhance the diagnosis and monitoring of Crohn’s disease and other gut disorders.

## Materials and methods

### Clinical study design

To investigate opportunities for improved diagnostics and advanced characterisation of gut function in Crohn’s disease, we recruited 20 healthy volunteers and 38 Crohn’s patients to participate in a clinical study of gut function using TFS and the L:M test. All experiments were performed at St. Mary’s Hospital, London, UK.

A sample size of 58 participants was chosen based on an estimated calculation of the number of participants required to reach a statistical power of 80%.^29^ As TFS has not been previously applied to studies of Crohn’s disease, and effect sizes were therefore unknown, this sample size calculation was based on published data reporting L:M ratio (LMR) values in healthy volunteers and Crohn’s disease patients. This calculation yielded a minimum sample size of 45 participants (15 healthy volunteers, 30 Crohn’s patients). To account for the potential for participant dropout and variability in disease severity (i.e. active vs inactive Crohn’s patients), we recruited a total of 58 participants (20 healthy volunteers, 38 Crohn’s patients). Full details of this sample size calculation can be found in the approved clinical study protocol.^29^

Of the 58 participants recruited to the study, datasets for two healthy volunteers and three Crohn’s patients were excluded from analysis. Healthy volunteers denoted as HV009 and HV012 were excluded from all analyses as one was later found to have liver disease (HV009) while the second was subsequently found to have not fasted prior to the study (HV012) (see Figure S1). Datasets for three Crohn’s patients (denoted as CD016, CD021 and CD037) were excluded from analysis due to errors in data collection that led to low-quality fluorescence datasets (see Figures S2 and S3). In addition, the dataset for one healthy volunteer (HV015) was excluded from some analyses, as data collection was terminated early (see further information in section ‘Fluorescence data analysis’).

Age and body mass index (BMI) were recorded for all participants. Clinical data – including Harvey-Bradshaw Index (HBI), faecal calprotectin (FCP), disease duration and extent (categorised according to the Montreal classification), and previous IBD therapy – were collected from all Crohn’s patients. Of note, there was no known upper GI disease activity in any patients (upper GI disease is likely to disproportionally affect gastric emptying rate compared to ileal or colonic disease only). A further two Crohn’s patients were excluded from the active versus the inactive analysis, as no FCP measurement was available (CD005 and CD008).

Patients with Crohn’s disease were divided into active Crohn’s (AC) and inactive Crohn’s (IC) groups based on their FCP level, with FCP > 150 µg/g indicating active disease. An FCP cut-off value of 150 µg/g was chosen in line with the International Organisation for the Study of Inflammatory Bowel Disease 2021 consensus, which recommends a target FCP ⩽ 150 µg/g as a treatment goal.^30^ We acknowledge that FCP is not highly specific, and that levels as high as 600 µg/g may still reflect only low-grade inflammation in some patients. Nevertheless, the STRIDE-II consensus defines FCP ⩽ 150 µg/g as indicative of endoscopic mucosal healing, and we adopted this threshold as a clinically relevant reference point for assessing intestinal permeability. Increased permeability is often seen as a sensitive marker in low-grade inflammation. This supports the use of this lower FCP threshold in our analysis. Importantly, one of the potential advantages of intestinal permeability assessment is its ability to detect subclinical inflammation, support risk stratification, and predict early relapse,^8,31^ which may occur even when FCP levels remain relatively low.

Table 1 shows the clinical information of the participants, for the final 18 healthy volunteers and 35 Crohn’s patients included in the study.

**Table 1.** Participant information.

Parameter/characteristic	Healthy controls, *n* = 18	CD patients, *n* = 35	Active CD (FCP > 150), *n* = 21	Inactive CD (FCP < 150), *n* = 12
Median age (range), years	28 (21–50)	39 (19–73)	40 (19–73)	33.5 (25–63)
Median BMI (range), kg/m^2^	25 (21.6–31)	25.75 (17.3–32)	25.95 (19–31.4)	25.6 (19–32)
Sex (male:female)	9:9	22:13	12:9	9:3
Mean disease duration, years		4.34	5.416	2.899
Age at diagnosis, %				
⩽16 (A1)		0	0	0
17–40 (A2)		65.7	66.7	58.3
>40 (A3)		34.3	33.3	41.7
Location, %				
Ileal (L1)		48.6	42.9	58.3
Colonic (L2)		2.86	4.76	0
Ileocolonic (L3)		45.8	52.4	41.7
Upper GI involvement (L4)		0	0	0
Behaviour, %				
Non-stricturing or penetrating (B1)		54.3	47.6	66.7
Stricturing (B2)		42.9	52.4	25
Penetrating (B3)		2.86	0	8.3
Perianal disease (p)		14.3	19	8.3
Mean FCP, µg/g		542	1012	49.4
Mean HBI		5.23	5.42	4.87
Previous inflammatory bowel disease therapy, %				
None		28.6	28.5	16.7
Biologics		34.2	28.5	41.7
Hemicolectomy/small bowel resection		11.4	14.2	0
Perianal procedures		5.71	4.7	8.3

Table shows demographic and clinical information of participants, including age, body mass index (BMI), sex balance and Montreal classification of disease state. Data are shown for participants included in study analysis (*n*_healthy_ = 18, *n*_Crohns_ = 35).

CD, Crohn’s disease; FCP, faecal calprotectin; HBI, Harvey-Bradshaw Index.

Prior to their assigned study day, participants were asked to fast overnight. Following arrival on the day of their experiment, participants gave written informed consent, and female volunteers were asked to take a urine pregnancy test (Alere™ hCG Easy, Abbott, IL, USA). A TFS probe was then attached to the participant’s forefinger, and fluorescence measurements were recorded as described in section ‘Transcutaneous fluorescence spectroscopy’. Following completion of their study day, all participants were given a kit to allow them to take an L:M test at home (see further details in section ‘Lactulose:mannitol test’). A flow chart illustrating the study protocol is presented in Figure S4.

The reporting of this study conforms to the Strengthening the Reporting of Observational Studies in Epidemiology statement for cross-sectional observational studies.^32^

### Transcutaneous fluorescence spectroscopy

**Figure 1.**
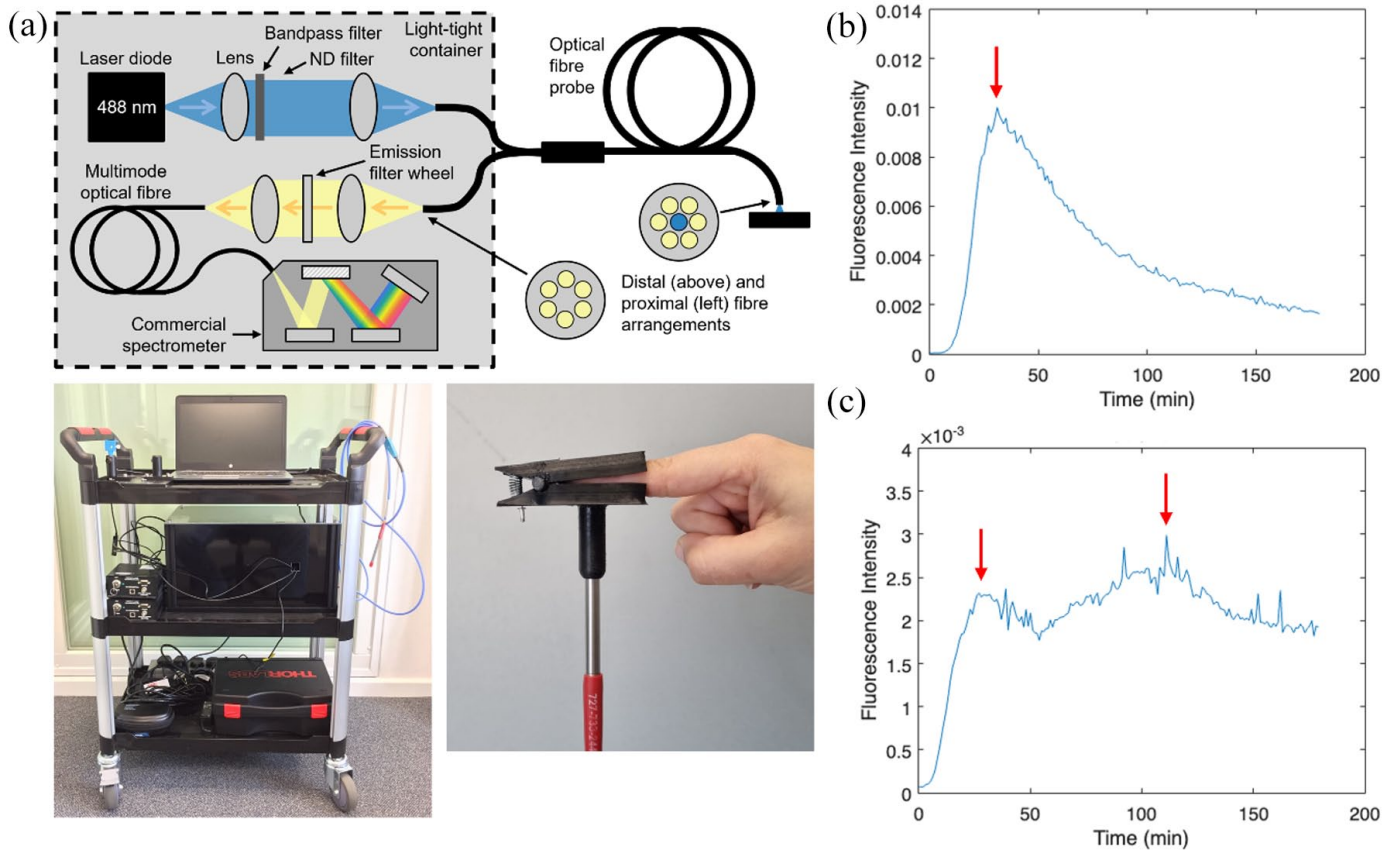
TFS system and example data. (a) Schematic diagram of optical system used for TFS (top) and photographs of full device (bottom left) and fingerclip for attaching fibre probe to skin (bottom right). (b) Example of fluorescence time course collected in one individual (CD019) showing a typical single-peaked fluorescence curve. (c) Example of fluorescence time course collected in one individual (CD001) showing a double-peaked fluorescence curve. Red arrows indicate peaks. TFS, transcutaneous fluorescence spectroscopy.

Transcutaneous fluorescence measurements were recorded using a fibre-optic spectrometer originally reported in Maurice et al.^22^ This system comprises a 488 nm laser source for excitation of fluorescence (Stradus 488-25, Vortran Laser Technology, CA, USA), a bifurcated fibre-optic probe (QR200-7-VIS-NIR, Ocean Optics, Duiven, The Netherlands) to allow measurement of fluorescence signals at the skin, and a compact spectrometer for detection/quantification of the fluorescence and reflectance signals collected by the probe (Figure 1(a)). A detailed description of this device (including the hardware, optical safety considerations and data collection procedures) can be found in Maurice et al.^22^ and Lett et al.^23^

To perform transcutaneous fluorescence measurements, the fibre-optic probe was attached to the participant’s forefinger using a 3D-printed, spring-loaded finger-clip (Figure 1(a)). This fingerclip ensured that the fibre probe remained in gentle but firm contact with the skin for the duration of the experiments. After attaching the fingerclip, fluorescence readings were initiated, and the participant was then asked to ingest a 100 ml solution of aqueous fluorescein (concentration = 5 mg/ml; total mass of fluorescein = 500 mg). Fluorescein was purchased from Imperial College Healthcare NHS Trust Pharmacy (Anatera, Alcon, Novartis Pharmaceuticals, Basel, Switzerland). Fluorescence readings were then taken at 1-min intervals for a total of 3 h, resulting in the collection of one fluorescence versus time curve for each participant (see example datasets in Figure 1(b) and (c) and datasets for all participants in Figures S1–S3).

### Fluorescein as a permeability probe

Fluorescein sodium is a widely used agent for assessing intestinal permeability in ex vivo animal models and in vitro studies.^33,34^ It has a molecular weight of 376 g/mol, which is similar to that of lactulose (342 g/mol). Being a hydrophilic molecule with limited transcellular transport, its absorption primarily occurs via the paracellular route through tight junctions.^35^ Owing to its strong fluorescence emission properties, fluorescein is readily detectable via spectroscopy, making real-time, non-invasive assessment of intestinal permeability a possibility.

Fluorescein has a good safety profile, particularly when administered orally. Using a 1 g oral dose, two large-scale studies involving 1019 and 1787 patients reported no severe adverse events and only mild side effects such as nausea or abdominal discomfort in <1% and 1.7% of participants, respectively.^36,37^ To our knowledge, only two cases of anaphylaxis associated with oral fluorescein have been reported in the literature.^38,39^

In this study, an oral solution containing 500 mg fluorescein (half the dose used in the above studies) was administered by a qualified clinician who also monitored for any adverse events during the experiment. An Epipen^©^ (Meridian Medical Technologies, MD, USA) was available for use in the event of allergic reactions. Any adverse events and serious adverse events were to be reported according to the study protocol.^29^

### Fluorescence data analysis

**Figure 2.**
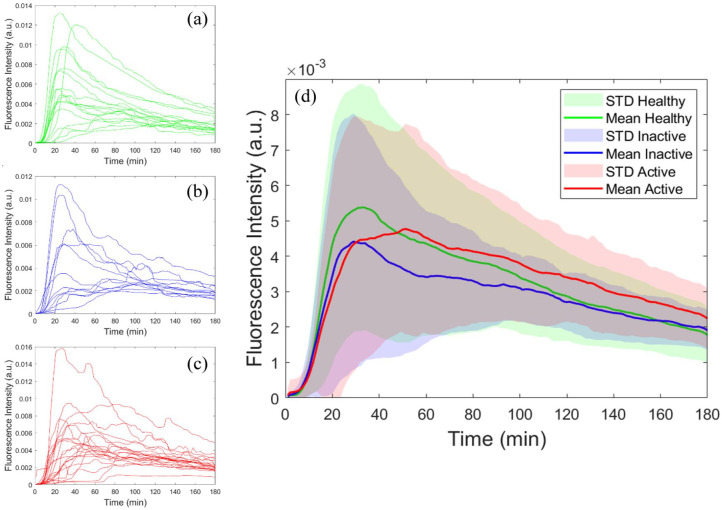
Individual and average fluorescence versus time curves. (a–c) Individual fluorescence versus time curves for all participants: (a) healthy volunteers, (b) inactive Crohn’s patients, (c) active Crohn’s patients. (d) Average fluorescence versus time curves for healthy volunteers (green), inactive Crohn’s patients (blue) and active Crohn’s patients (red). Solid lines represent mean fluorescence intensity values calculated across all participants in each group. Shaded areas represent error bars of ±1 STD. STD, standard deviation.

When interrogating the fluorescence versus time curves collected for each participant and the mean curves calculated in each cohort (i.e. healthy volunteers, active Crohn’s disease, inactive Crohn’s disease), we observed variability in both the maximum intensity and the time at which peak was reached (Figure 2 and Figures S1–S3). Thus, to assess whether transcutaneous fluorescence data could reveal differences in intestinal permeability and gastric emptying rate between healthy volunteers and Crohn’s disease patients, we extracted four key parameters from the TFS curves: (i) the time at which peak fluorescence intensity was reached (peak time); (ii) the area under the curve from the start of the measurement up to the peak time (area under the curve (AUC)); (iii) the AUC divided by the average gradient of the graph in the uptake region (i.e. from the start of the measurement up to the peak time) (AUC/slope); and (iv) the AUC multiplied by the peak time (AUC * peak time). Datasets were smoothed using a median filter (window width = 10) prior to calculation of these parameters to reduce the impact of noise.

For gastric emptying, our previous work in healthy volunteers demonstrated that later TFS peak times are indicative of slower gastric emptying (as measured by the paracetamol absorption test, a validated method for assessing liquid gastric emptying).^23^ In that study, two independent analysis approaches were used to assess gastric emptying using TFS: (i) direct measurement of the time to maximum (peak) fluorescence intensity and (ii) curve-fitting to estimate emptying kinetics. Measurements were performed following ingestion of a standardised liquid meal (milkshake) – consistent with standard clinical protocols for gastric emptying assessment – containing both fluorescein and paracetamol. That experimental design allowed for a detailed assessment of gastric emptying using a standardised (liquid) test meal and comparison against a clinical standard (paracetamol absorption test). By contrast, the current study involved fasted-state TFS measurements (using fluorescein dissolved in water) without an accompanying liquid challenge (test meal) or external reference (such as the paracetamol absorption test) for validation of gastric emptying measurements. As such, here we elected not to calculate gastric emptying times or rates directly. Instead, we used TFS peak time as a simple and pragmatic indicator of gastric emptying rate. We acknowledge, however, that this approach has limitations and did not provide an absolute quantification of gastric emptying (rather, it provides a relative indication).

For permeability assessment, AUC (measured up until the peak time) was chosen to reflect the total amount of fluorescein absorbed, and thus intestinal permeability. A higher fluorescence AUC indicates that a greater amount of fluorescein has passed from the gut into the bloodstream and thus infers higher intestinal permeability. AUC was measured from the start of measurements until the time at which the peak was reached (and not for the full duration of the curves) to reduce the effect of elimination. Faster elimination rates will act to reduce total AUC by generating a faster drop in fluorescence intensity in the later stages of the time course. To restrict the impact this had on our permeability estimation, we calculated AUC up to the peak time only. This acted to focus this metric on time periods dominated by uptake of fluorescein and limit the impact of time periods dominated by elimination.

However, AUC is also affected by gastric emptying rate: slower emptying delays fluorescein delivery to the intestine, during which time more dye is eliminated, leading to a lower AUC even if permeability is high. To reduce this confounding effect, we introduced two additional parameters: AUC/slope and AUC * peak time. AUC/slope accounts for the rate at which fluorescein reaches the small intestine, helping to decouple uptake amount from delivery speed. AUC * peak time scales the absorption curve by the time delay, compensating for potential underestimation of AUC due to prolonged gastric retention and dye elimination. These AUC parameters were previously validated in healthy volunteers,^24^ where both AUC/slope and AUC * peak time detected experimentally induced changes in intestinal permeability.

While the observed fluorescence versus time curves typically exhibited the form shown in Figure 1(b) (with an increase up to a single peak followed by a steady decay), some datasets exhibited multiple peaks (see Figure 1(c) and Figures S1–S3). For this reason, we measured the above four parameters (peak time, AUC, AUC/slope, AUC * peak time) for both the first peak and the ultimate peak (i.e. the maximum point) in the data. All fluorescence parameters were then used to explore differences between groups and correlation with L:M parameters (see further details in section ‘Lactulose:mannitol test’).

One dataset (HV015) was excluded from mean curve calculation (Figure 2(d)) due to early termination of data collection, which introduced bias at later time points. However, since a peak was clearly identifiable (see Figure S1), this dataset was included in AUC and peak time analyses.

### Lactulose:mannitol test

Participants were provided with an L:M test kit to perform at home. Participants were asked to fast for a minimum of 6 h prior to taking the L:M test. Participants provided a baseline urine sample and then completely voided their bladders before ingesting a 200 ml aqueous solution containing 10 g of lactulose and 5 g of D-mannitol (Stratech Scientific Limited, Ely, UK). This was followed by the consumption of 300 ml of water. Urine collection was performed over the following 6 h (using 24-h urine collection bottles). During this 6-h period, participants were instructed not to consume any food or drink apart from 200 ml of water after 3 h. At the end of the 6-h collection, the full urine sample was returned to the study team. A total of 16 healthy volunteers, 9 active Crohn’s patients and 10 inactive Crohn’s patients completed L:M tests and returned urine samples.

The total volume of urine collected was measured (using the scale on the side of the urine collection bottles). Baseline and L:M urine samples were aliquoted and stored in a −80°C freezer until analysis. Concentrations of lactulose and mannitol were measured using liquid chromatography – tandem mass spectrometry (LC-MS/MS). Full details of the LC-MS/MS method are provided in the Supplemental Methods.

The percentage recovery of lactulose and mannitol in urine (%L and %M, respectively) were calculated using Equations (1) and (2):

(1) $$\%L=100\times \left(V\times \frac{C_{L}}{D_{L}}\right)$$

(2) $$\%M=100\times \left(V\times \frac{C_{M}}{D_{M}}\right)$$

In these equations, *V* represents the total volume of urine collected, *C_L_* and *C_M_* represent the urine concentrations of lactulose and mannitol, respectively, and *D_L_* and *D_M_* represent the administered doses of lactulose and mannitol, respectively (10 g for lactulose and 5 g for mannitol). The LMR was then calculated according to Equation (3)^40^:

(3) $$LMR=\frac{\%L}{\%M}$$

### Statistical analysis of TFS and L:M data

We performed statistical analysis to investigate whether the fluorescence and L:M parameters provided discrimination between disease states and whether there was a correlation between fluorescence and L:M parameters.

Participants were categorised into three groups: Healthy (H, *n* = 18); Active Crohn’s disease (AC, *n* = 21) and Inactive Crohn’s disease (IC, *n* = 12). TFS parameters analysed were peak time, AUC, AUC/slope and AUC * peak time. Urine sugar permeability assay parameters investigated were percentage lactulose and mannitol excretion (%L and %R, respectively), and LMR.

Quantile–Quantile (Q–Q) plots and Shapiro–Wilk tests were used to assess if any cohorts followed normal distributions. Where data were found to be non-normally distributed, Kruskal–Wallis tests were used to compare the difference in median across the three cohorts. Where significant differences were present, to identify which pairs of groups differed, one-sided Dunn’s tests were performed for non-parametric post hoc analysis. Rank biserial correlation (*r*) was then calculated to assess effect size. Benjamini–Hochberg (BH) correction was applied to control for inflated false discovery rates across multiple comparisons, reducing type I error whilst retaining reasonable power. BH correction was preferred to more conservative methods (e.g. Bonferroni) as it is better suited for limited sample sizes.

To investigate the correlation between fluorescence and L:M parameters, we first plotted scatter graphs of fluorescence versus L:M values and calculated linear regression trend lines. Second, we calculated Spearman’s rank correlation coefficients (*r_s_*) to quantify the strength of correlation and to investigate statistical significance.

## Results

### Transcutaneous detection of orally ingested fluorescein

No adverse events were reported throughout the study.

Following oral ingestion of aqueous fluorescein, we observed transcutaneous fluorescence signals at the fingertip in all participants (Figures S1–S3). Fluorescence versus time curves typically exhibited a rise to a peak over the first 20–60 min followed by a steady decay back towards zero (as shown in Figure 1(b)), representing uptake of fluorescein from the gut into the bloodstream followed by elimination of fluorescein (through both renal and hepatic pathways). In some cases, double-peaked fluorescence versus time curves were observed (see Figure 1(c) and Figures S1–S3). This effect was tentatively attributed to gastric migrating motor complex activity during the fasting phase, where 45–60 min of muscular quiescence is followed by frequent phasic contraction activity, resulting in cyclical ‘dumping’ of gastric content into the intestine.^41^ As explained in section ‘Fluorescence data analysis’, we calculated AUC parameters based on both the first and ultimate (maximum) peak in the data to (i) ensure that our analysis considered the total amount of fluorescein absorbed and (ii) investigate the possibility for rapid analysis (by taking earlier measurements at the first peak in the data).

Figure 2 shows the fluorescence versus time curves recorded in individual participants (separated according to disease state, Figure 2(a)–(c)) as well as the average (mean) curves calculated across all participants in each cohort (Figure 2(d)). The average curves for healthy participants and inactive Crohn’s patients exhibit similar shapes, with the peak reached at approximately 30–35 min. The curve for active Crohn’s disease, on the other hand, exhibits a slower rise, reaching peak at approximately 50–55 min.

Interestingly, the mean curves for healthy participants, inactive Crohn’s and active Crohn’s patients have similar peak intensities (Figure 2(d)). This would at first glance suggest similar permeability across all groups, which would contradict established evidence linking Crohn’s disease with impaired gut barrier function. However, as reported previously, a later fluorescence peak time indicates slower gastric emptying^23^ and hence suggests that a greater proportion of fluorescein would have been eliminated from the bloodstream by the time that peak was reached (assuming equal elimination rates across participants). Thus, the later peak time observed in active Crohn’s disease also acts to reduce the peak intensity (due to the longer period of elimination), leading to similar maximum values even if more fluorescein crosses the gut barrier in Crohn’s disease than in healthy participants.

Furthermore, variability across participants can also partially explain the similarity in peak intensities. We observed considerable heterogeneity in peak intensities and peak times across participants (Figure 2(a)–(c) and Figures S1–S3), which we attributed to inter-participant variability in gut barrier function and gastric emptying rate (e.g. due to differences in diet, alcohol consumption, and exercise levels^42–46^). This inter-participant variability acted to mask differences across cohorts to a certain degree. Nonetheless, Figure 2(d) demonstrates qualitative differences between groups – in particular, between active Crohn’s disease and healthy or inactive states – in terms of both peak time and AUC.

### Transcutaneous spectroscopy provides non-invasive detection of changes in intestinal permeability and gastric emptying rate

**Figure 3.**
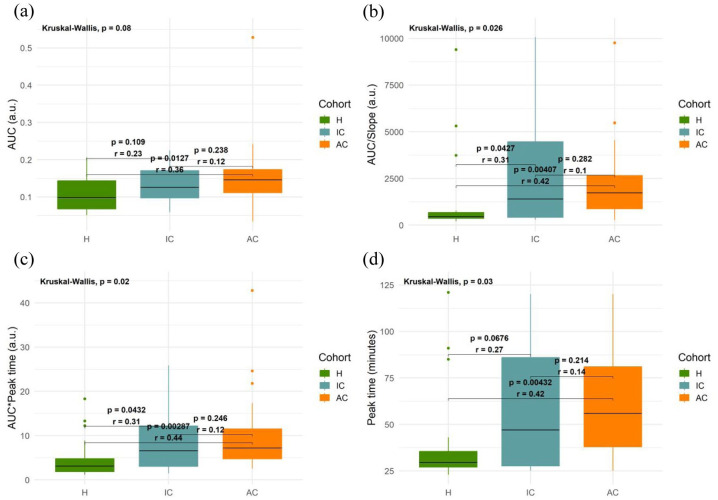
Box and whisker plots showing TFS-derived parameters for all participant groups. (a) AUC. (b) AUC/slope. (c) AUC * peak time. (d) Peak time. Values calculated at the ultimate peak in the fluorescence versus time curves. H – healthy (*n* = 18); IC – inactive Crohn’s (*n* = 12); AC – active Crohn’s (*n* = 21). Horizontal lines represent median values; lower and upper bounds of boxes represent 25th and 75th percentiles, respectively; whiskers extend to the most extreme data points (excluding outliers); solid circles represent outliers (defined as points that fell below the 25th percentile or above the 75th percentile by more than 1.5 times the interquartile range). Outliers were plotted for visualisation purposes only. All calculations and statistical tests were performed based on full datasets. Inset numbers represent *p*-values from post hoc Dunn’s tests performed between groups and corresponding rank biserial correlation effect sizes (*r*). TFS, transcutaneous fluorescence spectroscopy.

To further examine the qualitative differences observed in the fluorescence versus time curves collected in healthy volunteers, inactive Crohn’s patients and active Crohn’s patients, we extracted the peak time and three AUC parameters (AUC, AUC/slope, AUC * peak time – see section ‘Fluorescence data analysis’) from all individual datasets. Figure 3 demonstrates that these parameters exhibited differences across the different participant groups (when calculated at the ultimate peak in the data).

Shapiro–Wilk tests rejected the assumption of normality across all assessed parameters. Non-parametric tests were therefore used. Q–Q plot and Shapiro–Wilk test results are shown in Figures S5–S7.

For AUC parameters (which are indicative of intestinal permeability^24^), the median values for all three parameters (AUC, AUC/slope and AUC * peak time) were highest in active Crohn’s disease (AC) and lowest in healthy controls (H) (Figure 3(a)–(c)). The Kruskal–Wallis test revealed significant differences in AUC/slope (*p* = 0.026) and AUC * peak time (*p* = 0.02). AUC did not exhibit a significant difference (*p* = 0.08). Given the small number of participants, a post hoc pair-wise Dunn’s test was still performed for AUC.

After BH correction, significant differences were found between H and AC in all three permeability parameters, with medium effect size observed in all (*p* = 0.0127, *r* = 0.36 for AUC; *p* = 0.00407, *r* = 0.42 for AUC/slope; *p* = 0.00287, *r* = 0.44 for AUC * time). Differences in active versus inactive and healthy versus inactive were not found to be statistically significant apart from H versus IC in AUC * peak time (*p* = 0.0432, *r* = 0.31) (Figure 3(a)–(c)).

Peak time, an indicator of gastric emptying rate,^23^ was found to be highest in active Crohn’s patients and lowest in healthy volunteers (*p* = 0.00432, *r* = 0.42) (Figure 3(d)). Other paired tests did not reach statistical significance.

We also calculated AUC and peak time parameters at the first peak in the data (see Figures S8 and S9). In that case, similar trends were observed but with generally lower significance levels.

These results suggest both increased intestinal permeability and slower gastric emptying in Crohn’s disease as compared to a healthy population. While both increased permeability^3–8^ and reduced gastric emptying rate^9–14^ have been reported in Crohn’s disease previously, to the best of our knowledge, this represents the first report of simultaneous assessment of permeability and gastric emptying using a single test. Furthermore, TFS enables rapid assessment of permeability and gastric emptying using an almost completely non-invasive measurement protocol. No biological samples are required, and data can be analysed immediately upon collection, allowing rapid reporting of results. Thus, this suggests significant potential of this technology for improved monitoring and diagnosis of Crohn’s disease (e.g. to assess therapy responses and to offer early detection of relapse) and for advanced study of gut function in Crohn’s and other GI disorders (i.e. to simultaneously assess multiple facets of gut health/function).

### TFS permeability parameters exhibit qualitative correlation with the L:M test

A subset of participants – 16 healthy volunteers, 9 active Crohn’s and 10 inactive Crohn’s patients – also completed the lactulose:mannitol (L:M) test after their TFS study visit. Although the L:M test was intended to be performed within 1 week of TFS, delays of up to 116 days occurred in some cases. This time delay likely introduced variability in gut function between tests, particularly in Crohn’s patients whose treatment status or disease activity may have changed. Combined with the small sample size, these factors limit interpretability. Notably, while increased permeability on the L:M test is widely reported in active Crohn’s disease, our study did not replicate this finding. Given these limitations, our L:M data should be viewed as exploratory.

**Figure 4.**
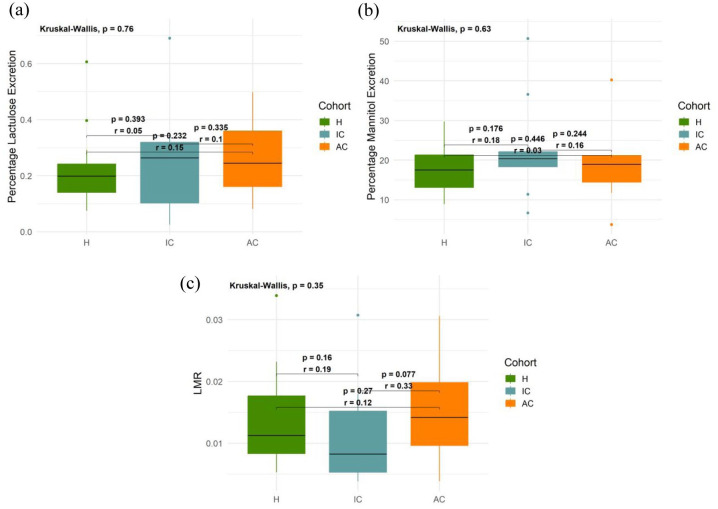
Box and whisker plots showing L:M parameters for all participant groups. (a) Percentage lactulose recovery (%L). (b) Percentage mannitol recovery (%M). (c) LMR. H – healthy (*n* = 16); IC – inactive Crohn’s (*n* = 10); AC – active Crohn’s (*n* = 9). Horizontal lines represent median values; lower and upper bounds of boxes represent 25th and 75th percentiles, respectively; whiskers extend to the most extreme data points (excluding outliers); solid circles represent outliers (defined as points that fell below the 25th percentile or above the 75th percentile by more than 1.5 times the interquartile range). Outliers were plotted for visualisation purposes only. All calculations and statistical tests were performed based on full datasets. Inset numbers represent *p*-values from Dunn’s tests performed between groups and rank biserial correlation effect sizes (*r*). LMR, Lactulose:mannitol ratio.

Across participant groups, trends in percentage lactulose recovery and LMR appeared broadly consistent with TFS-derived AUC parameters (Figure 4(a) and (c) versus Figure 3(a)–(c)), although no statistically significant differences were detected between groups (*p* > 0.05) with %L or LMR. No clear trends were observed in percentage mannitol recovery (Figure 4(b)).

**Figure 5.**
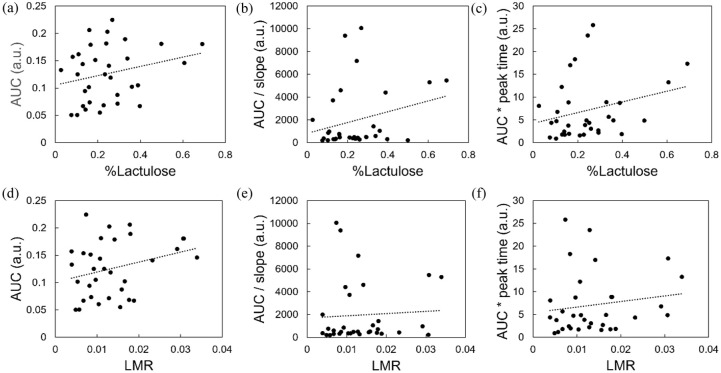
TFS versus L:M correlation analysis – scatter plots. Graphs show TFS-derived AUC parameters (calculated at ultimate peak) against percentage lactulose recovery (%L; a–c) and against lactulose:mannitol ratio (LMR; d–f). (a, d) AUC. (b, e) AUC/slope. (c, f) AUC * peak time. Dotted lines represent linear regression trend lines. LMR, Lactulose:mannitol ratio; TFS, transcutaneous fluorescence spectroscopy.

Scatter plots comparing TFS parameters (AUC, AUC/slope and AUC * peak time) against both %L and LMR revealed weak positive trends (Figure 5), but correlations were not statistically significant (Spearman’s rank correlation coefficient, *r_s_* ≈ 0.24–0.26; *p* > 0.13; Table 2). While not conclusive, these observations may suggest potential alignment between TFS and the L:M test, which is plausible given that TFS measures permeation of fluorescein – a single agent with molecular weight similar to lactulose.

**Table 2.** TFS versus L:M correlation analysis – statistical parameters.

Parameter	AUC	AUC/slope	AUC * peak time
%L	*r*_s_ = 0.247 *p* = 0.158	*r*_s_ = 0.217 *p* = 0.217	*r*_s_ = 0.261 *p* = 0.136
LMR	*r*_s_ = 0.244 *p* = 0.164	*r*_s_ = 0.100 *p* = 0.572	*r*_s_ = 0.157 *p* = 0.375

Spearman’s rank correlation coefficients (*r*_s_) and corresponding *p*-values for correlation of TFS-derived AUC parameters (AUC, AUC/slope, AUC * peak time) with %L and LMR.

LMR, Lactulose:mannitol ratio; TFS, transcutaneous fluorescence spectroscopy.

Interestingly, weak positive trends were observed between fluorescence parameters and %M recovery (Figure S10), though none reached statistical significance and correlation coefficients were lower than those for %L or LMR (i.e. compare Table 2 against Table S1). While this may appear counterintuitive – since mannitol is generally absorbed irrespective of gut integrity – it may point to limitations of the L:M test. Mannitol is intended to normalise lactulose absorption by accounting for physiological variability (e.g. gastric emptying, renal/hepatic clearance and dilution). However, in this study, %L and %M appeared positively associated (Figure S11), suggesting that mannitol may obscure permeability changes rather than correct for them. This raises the possibility that single permeability probes could serve as more reliable markers of intestinal permeability than LMR, provided appropriate controls are in place. This is consistent with suggestions from recent studies (e.g. Khoshbin et al.^47^).

## Discussion

We applied TFS and the L:M test to a study of gut function in Crohn’s disease. Our TFS results demonstrate changes in both gut barrier function and gastric emptying rate across healthy volunteers and inactive and active Crohn’s disease patients. Importantly, this was achieved using a rapid, non-invasive measurement protocol.

Among the fluorescence-derived parameters investigated, AUC served as a direct measure of fluorescein uptake, and therefore of intestinal permeability. However, since delayed gastric emptying can reduce peak fluorescence intensity due to ongoing elimination, the additional derived parameters AUC/slope and AUC * peak time were introduced to account for kinetic variability. These adjustments were designed to reduce the confounding effect of gastric emptying rate on permeability measurements, enabling better physiological interpretation.

L:M parameters showed positive, though non-significant, associations with TFS parameters, suggesting that the novel TFS test may assess small intestinal permeability similarly to the L:M test. While these trends should be interpreted with caution, the direction is consistent with a shared underlying physiological process.

While changes in gut barrier function^3–8^ and gastric emptying^9–14^ in Crohn’s disease have been reported previously, these parameters have not previously been measured simultaneously using the same test/assay, nor have they been measured in a rapid, non-invasive manner. Indeed, variability in gastric emptying rate is typically considered as a confounder for measurements of gut barrier function and acts to induce uncertainty into measurements made with the L:M test (and other urinary sugar assays). Our results demonstrate that TFS can provide readouts of both factors simultaneously, inherently accounting for confounding effects through the collection of data with high time resolution.

Fluorescence-based techniques have been widely applied to studies of gut function in cellular and animal studies in the past, to assess numerous aspects of gut health in IBD and other conditions (e.g. Drewe et al.,^48^ Sadowski and Meddings^49^ and Fleming et al.^50^). A small number of in vivo human studies have reported assessment of intestinal permeability via measurement of urinary recovery of orally ingested fluorescent probes, for example, in coeliac disease^51^ and Crohn’s disease.^52^ Our previous work reported the application of TFS to provide non-invasive assessment of permeability^22,24^ and gastric emptying^23^ in healthy volunteers (with no requirement for biological sample collection). Crucially, to the best of our knowledge, this study represents the first report of non-invasive fluorescence sensing for the assessment of gut function in a human disease study.

Taken together, the above indicates that TFS provides opportunities both for advanced monitoring and diagnosis of Crohn’s disease (e.g. for non-invasive, in-home assessment of therapy responses) and advanced study of gut function in health and disease (through simultaneous measurement of multiple gut functional parameters). TFS has numerous advantages over existing methods (such as the L:M test) due to its rapid, non-invasive nature, and thus offers potential to significantly improve both clinical protocols for diagnosis and monitoring and patient experiences (in Crohn’s disease and elsewhere).

The most important limitation of this study is the delay between TFS and L:M measurements. We designed the study to perform these tests on separate days to ensure that there was no interaction between tests. We aimed for participants to perform L:M tests within 1 week of their TFS experiment, which would have minimised variability due to changes in diet, lifestyle and medication. However, due to difficulties in participant follow-up, not all participants performed L:M tests, and several returned tests after considerable delays (of up to approx. 3 months; maximum delay of 116 days).

It is well known that intestinal function exhibits daily variation and is affected by numerous lifestyle and dietary factors (including activity/exercise level, stress, alcohol intake, etc.).^42–46^ Among others, high stress, high sugar diet, strenuous activity and particularly alcohol are all known to increase small bowel permeability. Reduced disease activity from medical treatment, on the other hand, is expected to reduce or normalise intestinal permeability. These have likely introduced considerable variability into our urinary sugar analysis. For future studies, urinary sugar collection time slots should be pre-arranged within 1 week of TFS, before any experimental work takes place, or performed simultaneously to TFS. Patients should be given written advice on avoiding lifestyle factors that alter intestinal permeability. In addition, improved checks for confounders should take place (e.g. performing urinary alcohol tests on arrival, and asking participants to keep food and drink diaries). Indeed, we are incorporating these improved protocols into our ongoing work.

Furthermore, age, BMI and sex may all independently introduce variation into TFS readings. Table 3 briefly describes how each may affect TFS readings. Importantly, good quality evidence on how each of these factors affects permeability or gastric emptying is still lacking, and this is partly due to large individual variability and the presence of multiple confounders.

**Table 3.** Potential effects of demographic factors on TFS readings.

Demographic factor	Intestinal permeability effects	Gastric emptying/motility effects	Other effects
Age*	• Possible increase (with increasing age) • Reduced tight junction expression^53^ • Altered mucus production^54^ • Dysbiosis • Preserved in healthy elderly^55^	• Reduced gastric emptying rate (with increasing age) • Loss of enteric neurons^56^ • Change in migratory neuron complex^57^	• Altered fluorescein metabolism • Reduced liver volume and blood flow^58^ • Reduced number of nephrons
BMI	• Possible increase (with increasing BMI) • Dysbiosis^59^ • Results not consistent^60^	• Unclear • Conflicting evidence^61–63^	• Altered fluorescein distribution^64^ • Altered body tissue composition • Altered cardiovascular function • Increased adipose tissue depth • Reduced fluorescence signal
Sex	• Unclear • Conflicting evidence^65,66^	• Men generally have faster gastric emptying than women^67,68^	• Slimmer fingers in females may cause more fingerclip displacement and motion artefacts

* Healthy elderly do not show significant differences compared to younger adults.

TFS, transcutaneous fluorescence spectroscopy.

Further experiments should also assess gastric emptying in a fasting state in healthy individuals using an accepted/standardised test (such as the paracetamol absorption test) alongside TFS. This would permit further development of algorithms/expressions for gastric emptying rate based on TFS signals, thereby enabling clearer separation between gastric emptying and permeability in fasted individuals.

Despite these limitations, our results demonstrate that TFS reveals statistically significant differences between healthy volunteers and Crohn’s patients in terms of both intestinal permeability and gastric emptying rate (Figure 3). Moreover, we observed weak but positive associations between TFS AUC parameters and both %L recovery and LMR (Figure 5). While these correlations were not found to be statistically significant, they are nonetheless noteworthy due to the considerable delay between experiments. Indeed, the fact that trends were discernible despite this limitation suggests that a positive correlation may indeed exist in reality.

When considering clinical implementation, a few practical barriers remain. First, our current TFS device – which uses a laser for fluorescence excitation, a spectrometer for detection and a custom-made fibre-optic probe for light delivery and collection – remains relatively expensive (total cost of components ≈ £9k). However, unlike traditional L:M assays (which usually require expensive MS analysis), TFS utilises a reusable device, does not require sample collection and may permit automated analysis of data immediately after collection. This means that the cost per test is expected to reduce significantly over time (current consumables costs are on the order of £10 per experiment). Second, the current device setup requires participants to remain seated for the duration of data collection (typically 3–4 h), and signal quality is susceptible to motion artefacts. Thus, the development of wearable sensors, with improved motion compensation to enable greater participant/patient flexibility, will be crucial to allow wider application in clinical settings. Third, given its high degree of automation and simplicity in setup, we anticipate inter-operator variability in TFS to be minimal, though this has not been formally evaluated.

Our ongoing work is addressing these hurdles through the development of miniaturised handheld/wearable prototypes.^69^ These devices are designed to reduce costs and allow at-home testing, potentially supporting frequent non-invasive monitoring of Crohn’s or other GI diseases. Interestingly, it is noteworthy that some of the reasons intestinal permeability has not been adopted as a disease activity monitoring tool include cost and impracticality. TFS may overcome these barriers.

## Conclusion

In conclusion, we used a novel fluorescent technology –TFS – to non-invasively assess gut barrier function and gastric emptying in a clinical study of Crohn’s disease. Our results demonstrated that TFS-derived parameters related to barrier function and gastric emptying revealed significant differences between groups as well as qualitative concordance with the L:M test (a widely used assay for assessment of gut barrier function). Importantly, TFS allows rapid, non-invasive measurements and does not require the collection of biological samples. Furthermore, it permits simultaneous assessment of permeability and gastric emptying (using a single test), two important aspects of gut health in Crohn’s disease and many other disorders. Overall, this implies potential for significant clinical impact by allowing both improved diagnostics and more advanced study of disease processes.

## Supplemental Material

sj-pdf-1-tag-10.1177_17562848251361634 – Supplemental material for Non-invasive fluorescence sensing reveals changes in intestinal barrier function and gastric emptying rate in a first-in-human study of Crohn’s disease Supplemental material, sj-pdf-1-tag-10.1177_17562848251361634 for Non-invasive fluorescence sensing reveals changes in intestinal barrier function and gastric emptying rate in a first-in-human study of Crohn’s disease by Jonathan Gan, Qian Chen, Elena Monfort Sanchez, Nilanjan Mandal, Jiacheng Xu, Zixin Wang, Arjun Agarwal, Emmanuel Oluwatunmise, Pratik Ramkumar, Ash Salam, Elena Chekmeneva, María Gómez-Romero, Lynn Maslen, Sharmili Balarajah, Robert Perry, Karl King Yong, Jonathan Hoare, Nick Powell, James Alexander, James Avery, Hutan Ashrafian, Ara Darzi and Alex J. Thompson in Therapeutic Advances in Gastroenterology

sj-pdf-2-tag-10.1177_17562848251361634 – Supplemental material for Non-invasive fluorescence sensing reveals changes in intestinal barrier function and gastric emptying rate in a first-in-human study of Crohn’s disease Supplemental material, sj-pdf-2-tag-10.1177_17562848251361634 for Non-invasive fluorescence sensing reveals changes in intestinal barrier function and gastric emptying rate in a first-in-human study of Crohn’s disease by Jonathan Gan, Qian Chen, Elena Monfort Sanchez, Nilanjan Mandal, Jiacheng Xu, Zixin Wang, Arjun Agarwal, Emmanuel Oluwatunmise, Pratik Ramkumar, Ash Salam, Elena Chekmeneva, María Gómez-Romero, Lynn Maslen, Sharmili Balarajah, Robert Perry, Karl King Yong, Jonathan Hoare, Nick Powell, James Alexander, James Avery, Hutan Ashrafian, Ara Darzi and Alex J. Thompson in Therapeutic Advances in Gastroenterology

## Declarations

**Ethics approval and consent to participate:** Ethical approval was obtained through the UK Health Research Authority (HRA) following review by the London Bromley Research Ethics Committee (REC; HRA IRAS ID – 242462, REC reference – 18/LO/0714). All participants gave written informed consent prior to inclusion in the study, and all experiments were performed according to the approved clinical study protocol and in accordance with Good Clinical Practice (GCP) guidelines and the World Medical Association’s Declaration of Helsinki.

**Consent for publication:** All participants gave written informed consent for research findings and information collected in the study to be published.

**Competing interests:** A.J.T. is the inventor of a patent relevant to the use of transcutaneous fluorescence spectroscopy as a tool for non-invasive monitoring of gut permeability and gastric emptying rate, and has a licensing and consulting agreement with MediBeacon, Inc. related to this method. All other authors declare no competing interests.

**Availability of data and materials:** Data underlying the results presented in this paper are not publicly available at this time, but may be obtained from the corresponding author upon reasonable request.
