# Supplementary material for: Non-invasive fluorescence sensing reveals changes in intestinal barrier function and gastric emptying rate in a first-in-human study of Crohn’s disease
Source: Ther Adv Gastroenterol. 2025 Aug 13;18:17562848251361634. doi: 10.1177/17562848251361634 (PMC12357025; doi:10.1177/17562848251361634)
Supplement: sj-pdf-2-tag-10.1177_17562848251361634 – Supplemental material for Non-invasive fluorescence sensing reveals changes in intestinal barrier function and gastric emptying rate in a first-in-human study of Crohn’s disease [file sj-pdf-2-tag-10.1177_17562848251361634.pdf]

SUPPLEMENTARY INFORMATION

Non-invasive fluorescence sensing reveals changes in intestinal barrier function and gastric emptying rate in a first-in-human study of Crohn's disease

Jonathan Gan[1,2†], Qian Chen[1,2†], Elena Monfort Sanchez[1,2], Nilanjan Mandal[1,2], Jiacheng Xu[1,2], Zixin Wang[1,2], Arjun Agarwal[1,2], Emmanuel Oluwatunmise[1,2], Pratik Ramkumar[1,2], Ash Salam[3], Elena Chekmeneva[3], María Gómez-Romero[3], Lynn Maslen[3], Sharmili Balarajah[4,5], Robert Perry[4,5], Karl King Yong[5], Jonathan Hoare[2,5], Nick Powell[4,5], James Alexander[4,5,6], James Avery[1,2], Hutan Ashrafian[1,2], Ara Darzi[1,2], Alex J. Thompson[1,2]*

[1] The Hamlyn Centre, Institute of Global Health Innovation (IGHI), Imperial College London, Exhibition Road, South Kensington, London, SW7 2AZ, United Kingdom

[2] Department of Surgery & Cancer, St Mary's Hospital, Imperial College London, South Wharf Road, London, W2 1NY, United Kingdom

[3] National Phenome Centre, Section of Bioanalytical Chemistry, Department of Metabolism, Digestion and Reproduction, Imperial College London, Hammersmith Hospital Campus, IRDB Building, London, W12 0NN, UK

[4] Department of Metabolism, Digestion and Reproduction, Imperial College London, Hammersmith Hospital Campus, London, W12 0NN, UK

[5] Gastroenterology Department, Imperial College Healthcare NHS Trust, St Mary's Hospital, South Wharf Road, London, W2 1NY, United Kingdom

[6] IBD Unit, St Mark's National Bowel Hospital, Acton Ln, London, NW10 7NS

[†]These authors contributed equally to this work

*Corresponding author: alex.thompson08@imperial.ac.uk

This PDF file includes:

Supplementary Figures S1-S11

Supplementary Tables S1-S2

Supplementary Methods

Supplementary Figures

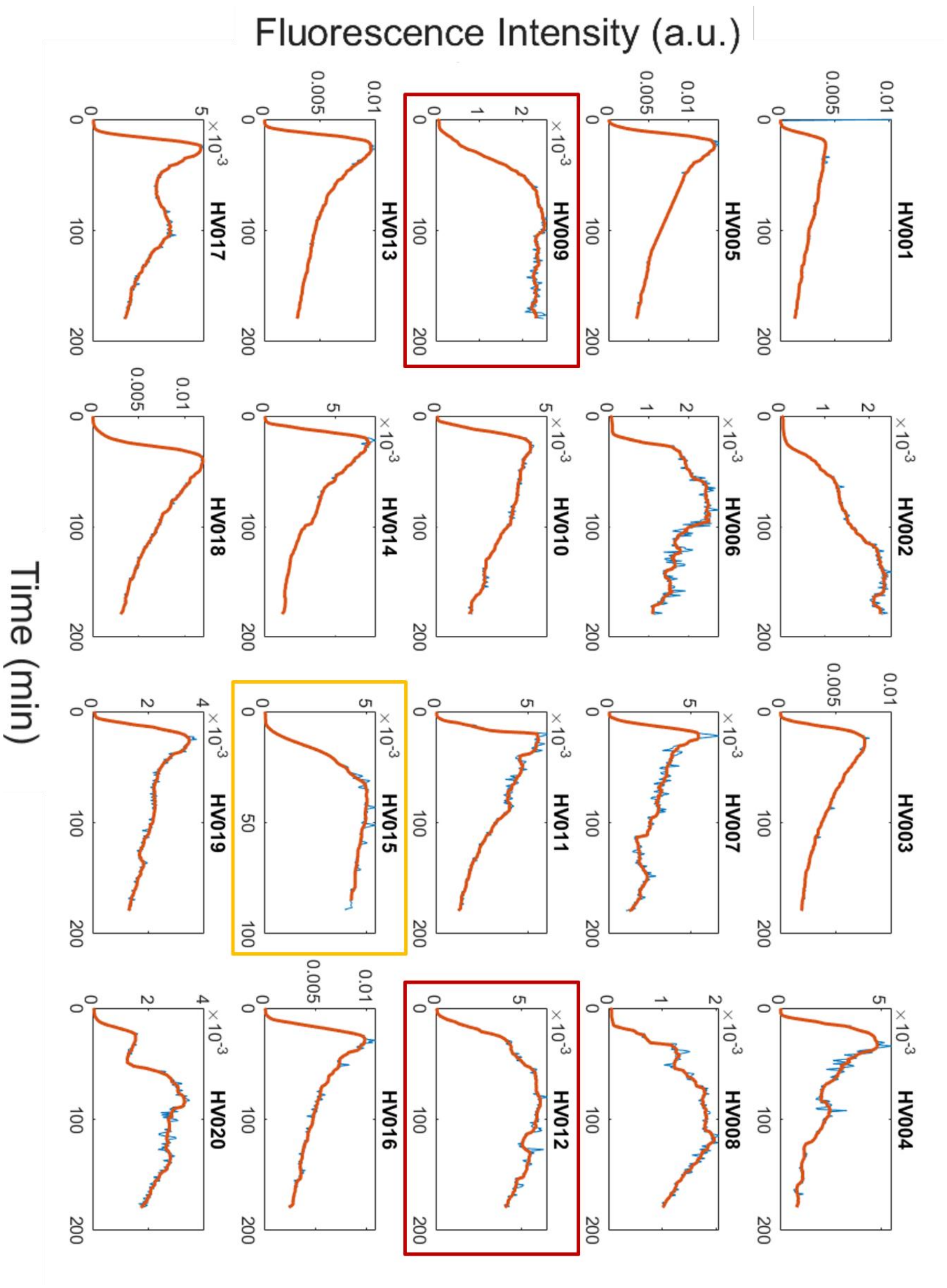

Figure S1. Fluorescence vs. time curves for all healthy volunteers. Blue lines represent raw datasets, red lines represent median-filtered curves. Red boxes indicate datasets (HV009 and HV012) that were fully excluded from analysis (see reasons for exclusions in section 2.1 of main article). Orange box indicates a dataset (HV015) that was excluded from calculation of mean fluorescence vs. time curve (Figure 2) but included in AUC analysis (Figure 3; see reasons for exclusion in sections 2.1 and 2.4 of main article).

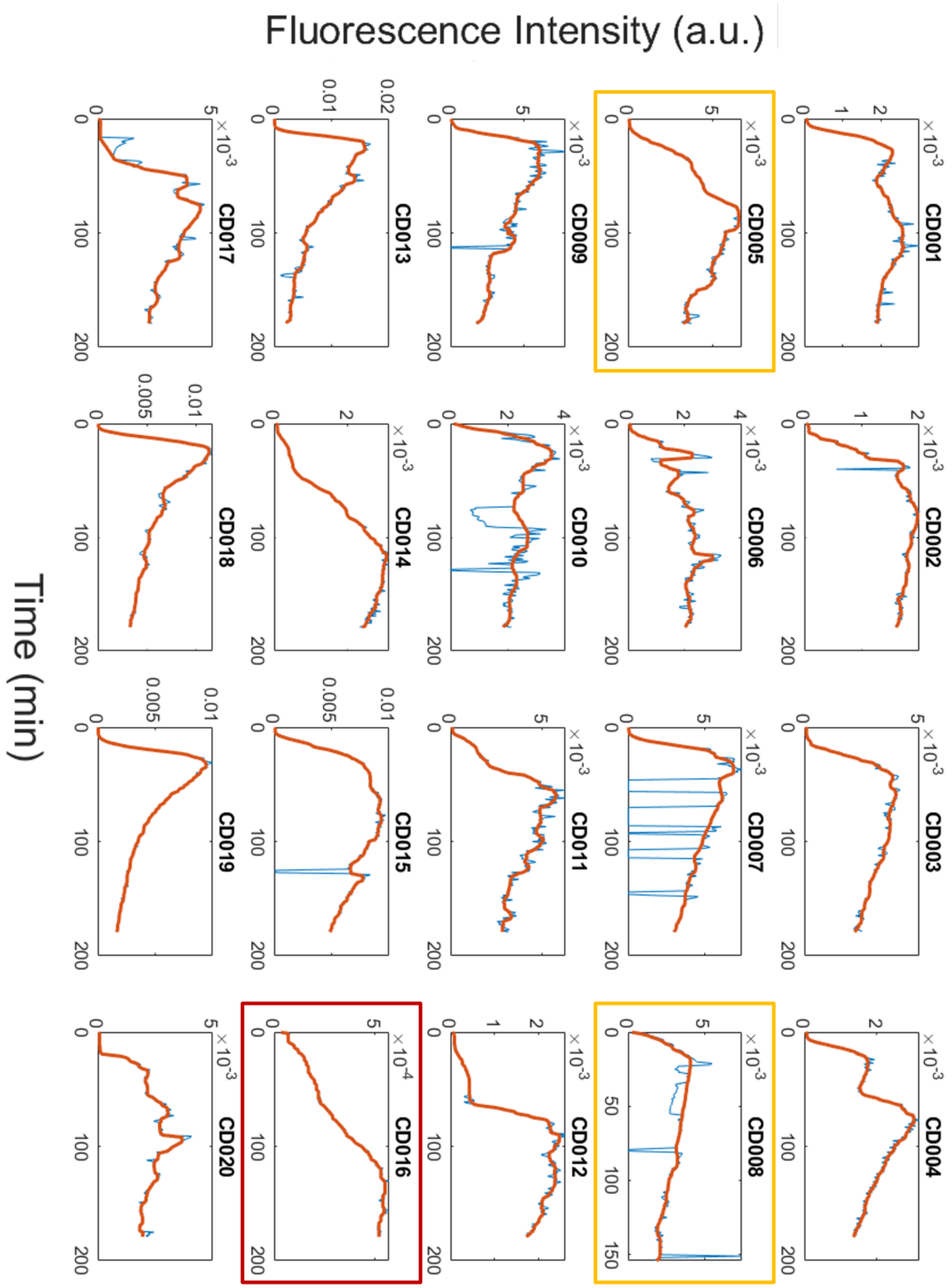

Figure S2. Fluorescence vs. time curves for Crohn's disease patients – participants CD001–CD020. Blue lines represent raw datasets, red lines represent median-filtered curves. Red box indicates a dataset (CD016) that was fully excluded from analysis (see reasons for exclusion in section 2.1 of main article). Orange boxes indicate datasets (CD005 and CD008) that were excluded from active vs. inactive analysis (as no FCP measurement was available; see section 2.1 of main article).

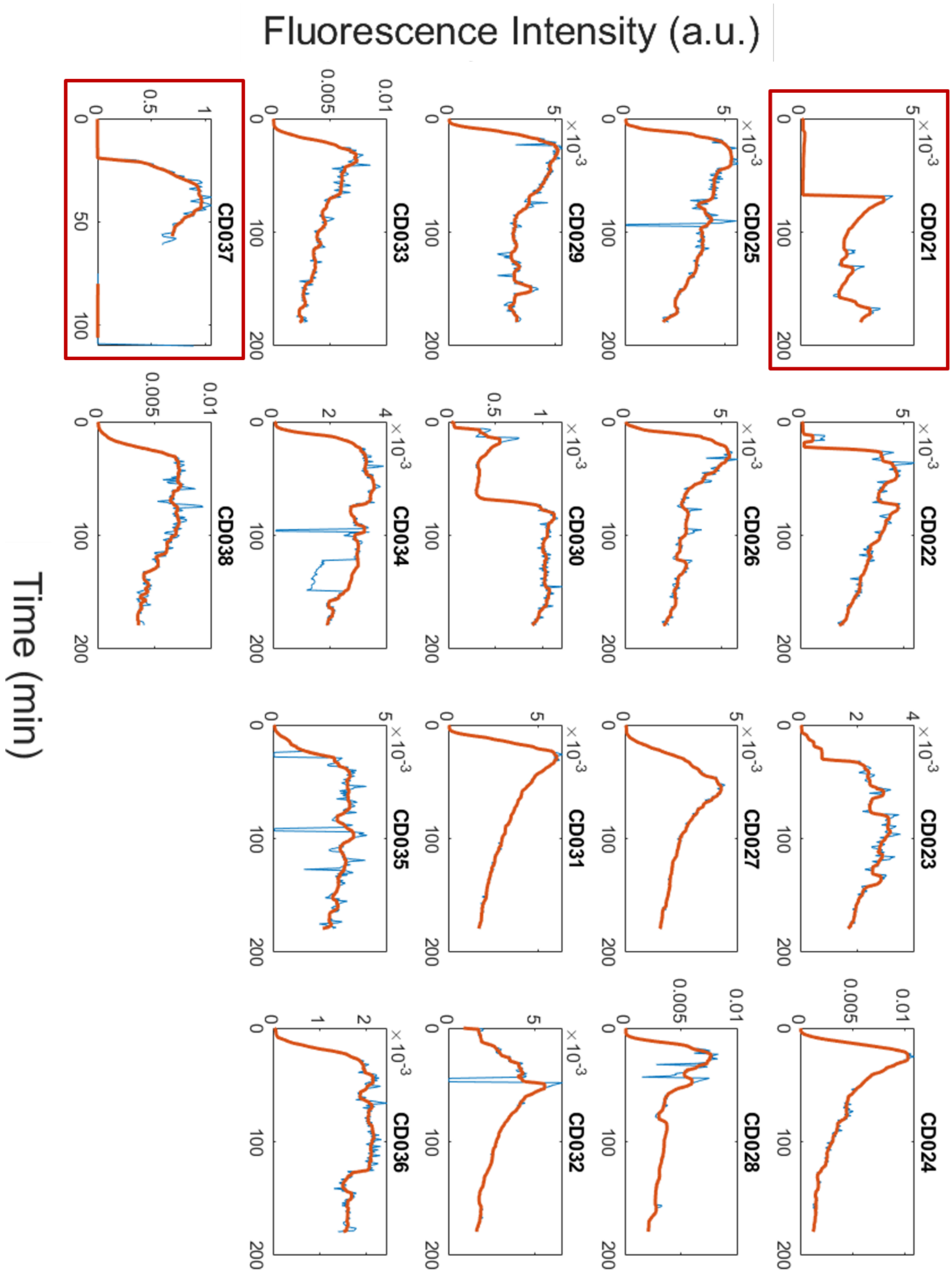

Figure S3. Fluorescence vs. time curves for Crohn's disease patients – participants CD021–CD038. Blue lines represent raw datasets, red lines represent median-filtered curves. Red boxes indicate datasets (CD021 and CD037) that were fully excluded from analysis (see reasons for exclusions in section 2.1 of main article).

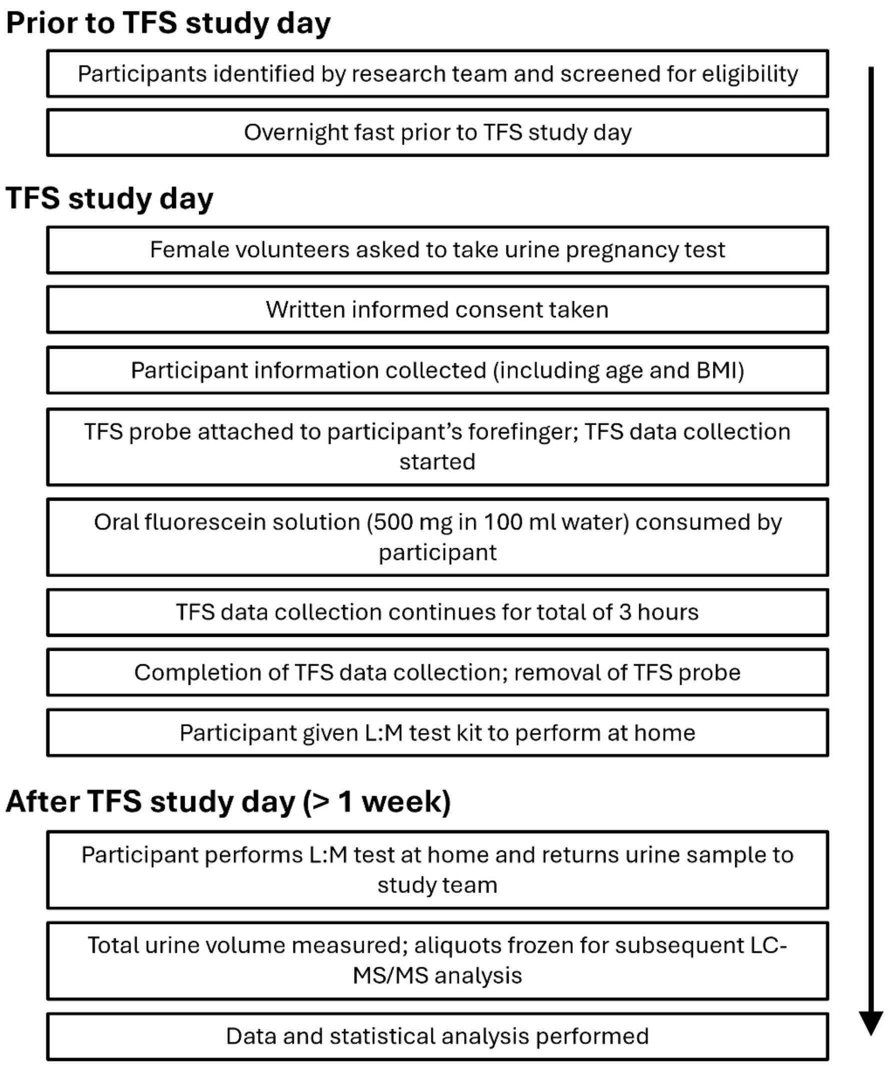

Figure S4. Flow chart outlining study protocol. TFS – transcutaneous fluorescence spectroscopy; L:M – lactulose:mannitol; LC-MS/MS – liquid chromatography – tandem mass spectrometry.

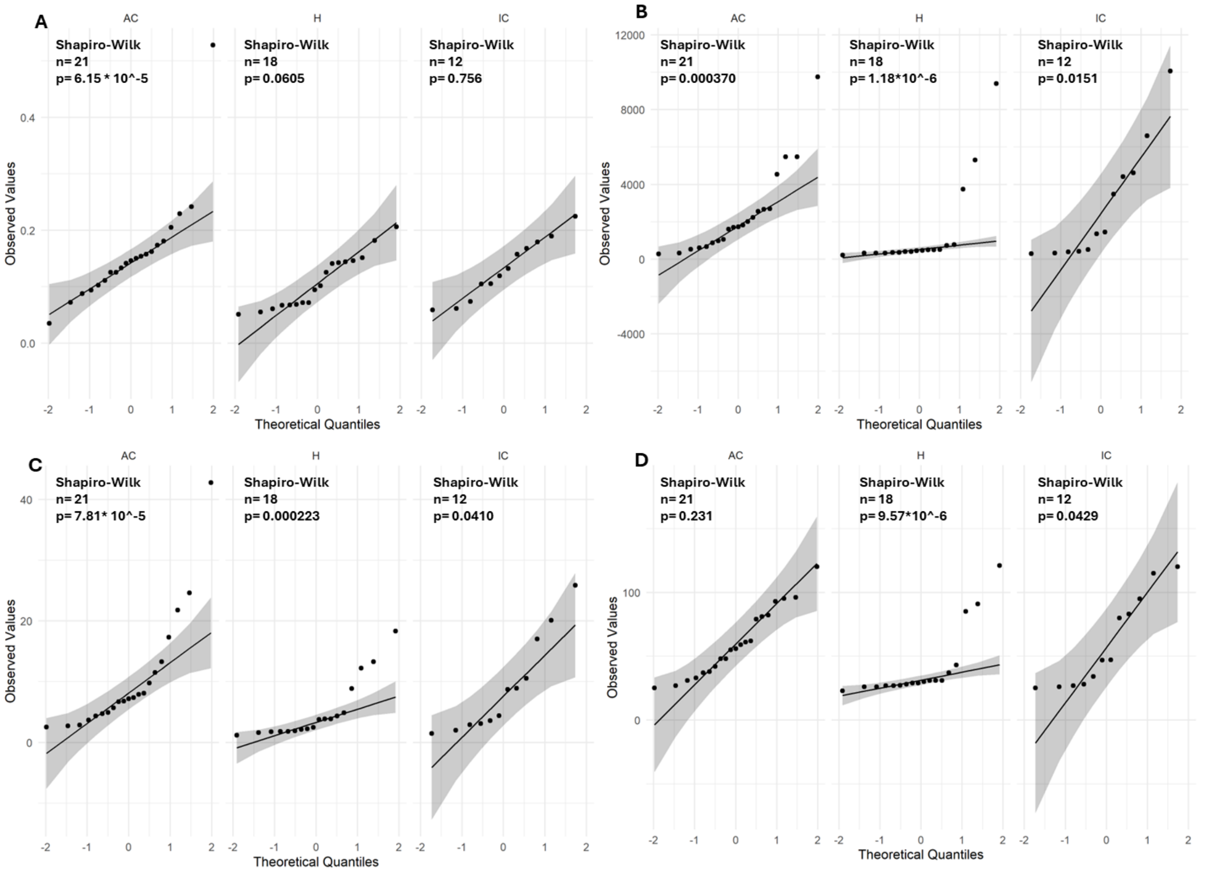

Figure S5. Q-Q plots for TFS derived parameters for all participant groups at ultimate peak. (**A**) AUC. (**B**) AUC / slope. (**C**) AUC * peak time. (**D**) Peak time. AC – Active Crohn's (n=21); H – Healthy (n=18); IC – Inactive Crohn's (n=12). *p*-values were calculated with the Shapiro-Wilk test to assess deviation from normality.

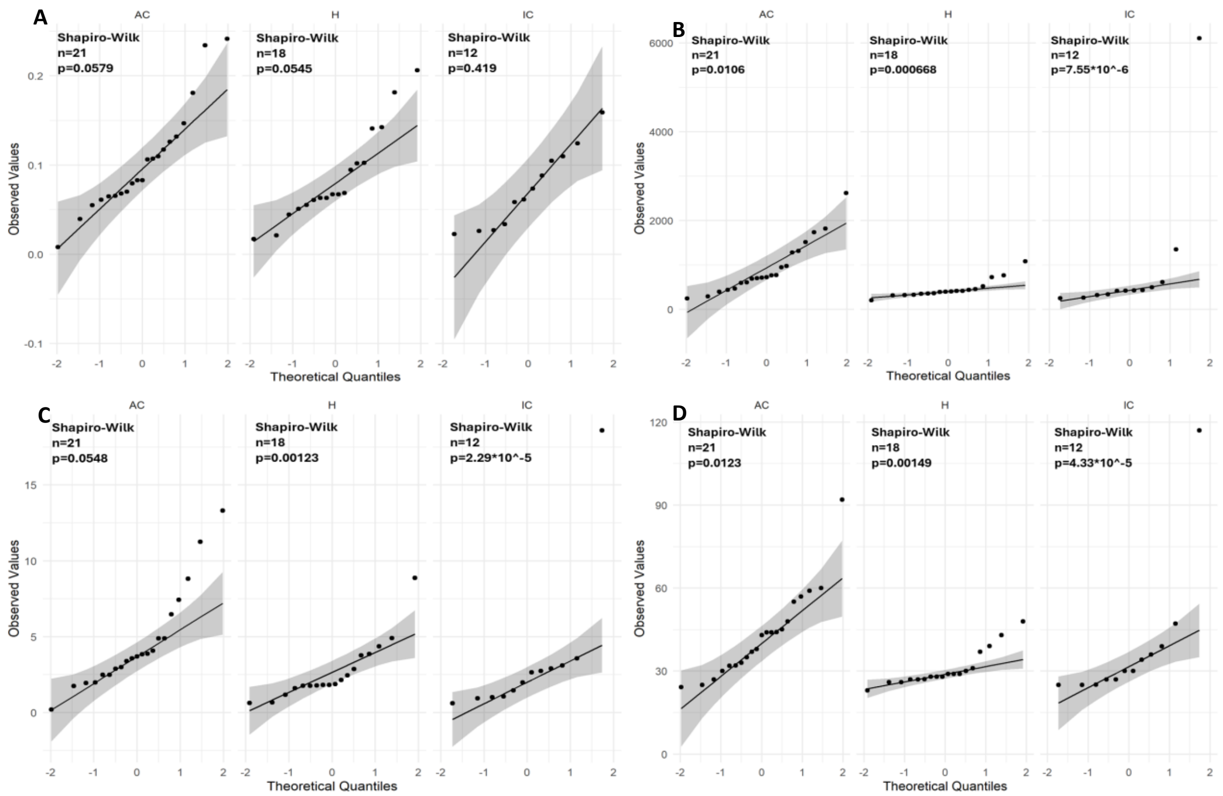

Figure S6. Q-Q plots for TFS derived parameters for all participant groups at first peak. (**A**) AUC. (**B**) AUC / slope. (**C**) AUC * peak time. (**D**) Peak time. AC – Active Crohn's (n=21); H – Healthy (n=18); IC – Inactive Crohn's (n=12). *p*-values were calculated with the Shapiro-Wilk test to assess deviation from normality.

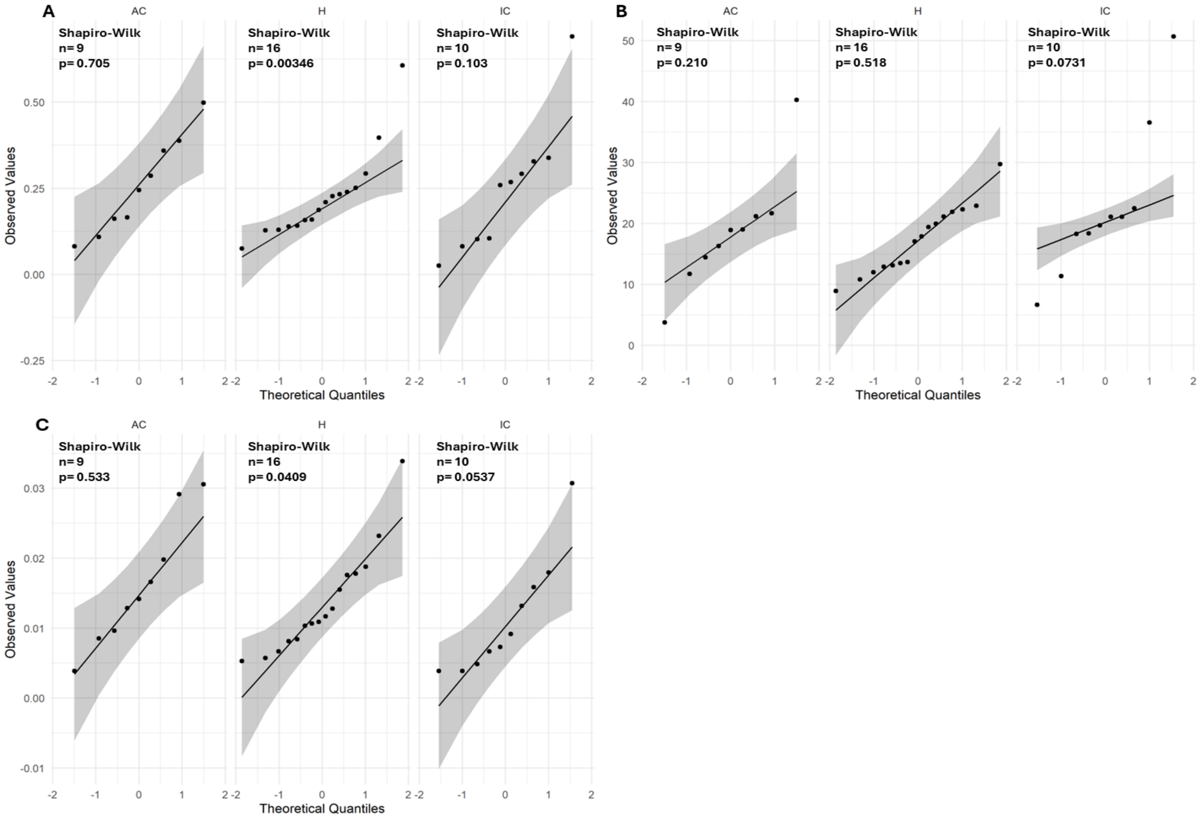

Figure S7. Q-Q plots for urinary sugar excretion for all participant groups. (**A**) Percentage lactulose recovery (%L). (**B**) Percentage mannitol recovery (%M). (**C**) Lactulose:mannitol ratio (LMR). AC – Active Crohn's (n=9); H – Healthy (n=16); IC – Inactive Crohn's (n=10). *p*-values were calculated with the Shapiro-Wilk test to assess deviation from normality.

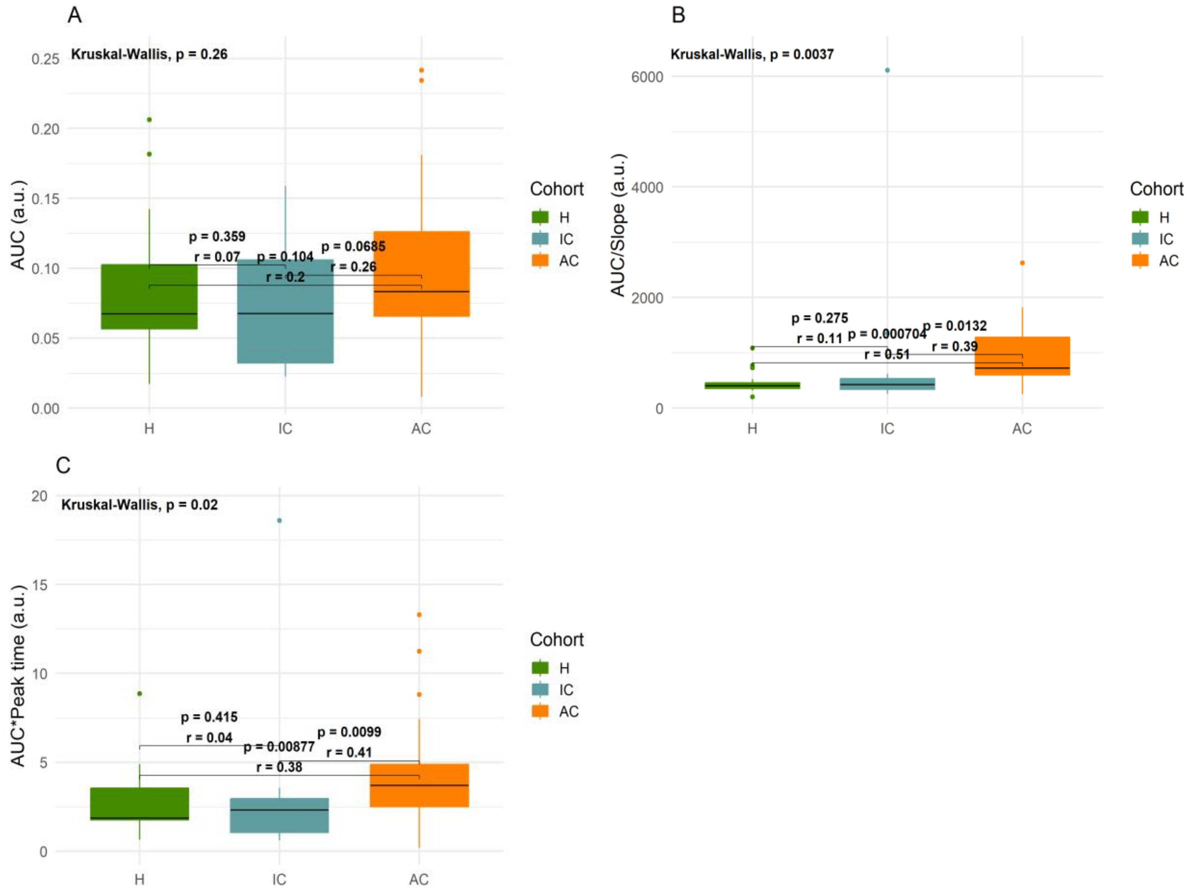

Figure S8. Box and whisker plots showing TFS-derived AUC parameters for all participant groups, calculated at the first peak in the fluorescence vs. time curves. (**A**) AUC. (**B**) AUC / slope. (**C**) AUC * peak time. H – healthy (n=18); IC – inactive Crohn's (n=12); AC – active Crohn's (n=21). Horizontal lines represent median values; lower and upper bounds of boxes represent 25th and 75th percentiles respectively; whiskers extend to most extreme data points (excluding outliers); solid circles represent outliers (defined as points that fell below the 25th percentile or above the 75th percentile by more than 1.5 times the interquartile range). All calculations and statistical tests were performed based on full datasets. Inset numbers represent *p*-values from post-hoc Dunn's tests performed between groups and corresponding rank biserial correlation effect sizes (*r*).

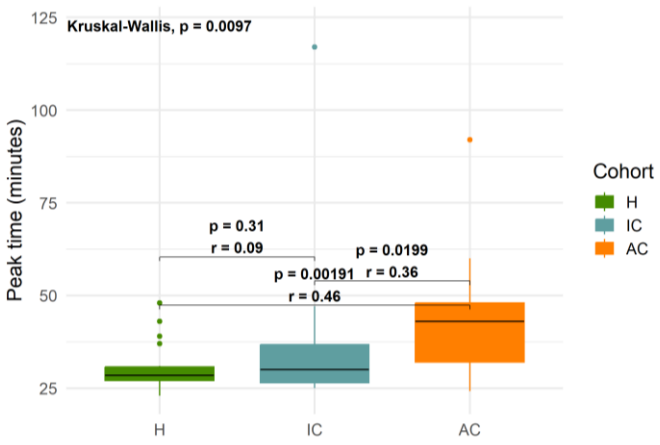

Figure S9. Box and whisker plot showing time of first peak in fluorescence vs. time curves for all participant groups. H – healthy (n=18); IC – inactive Crohn's (n=12); AC – active Crohn's (n=21). Horizontal lines represent median values; lower and upper bounds of boxes represent 25th and 75th percentiles respectively; whiskers extend to most extreme data points (excluding outliers); solid circles represent outliers (defined as points that fell below the 25th percentile or above the 75th percentile by more than 1.5 times the interquartile range). Outliers were plotted for visualisation purposes only. All calculations and statistical tests were performed based on full datasets. Inset numbers represent *p*-values from post-hoc Dunn's tests performed between groups and corresponding rank biserial correlation effect sizes (*r*).

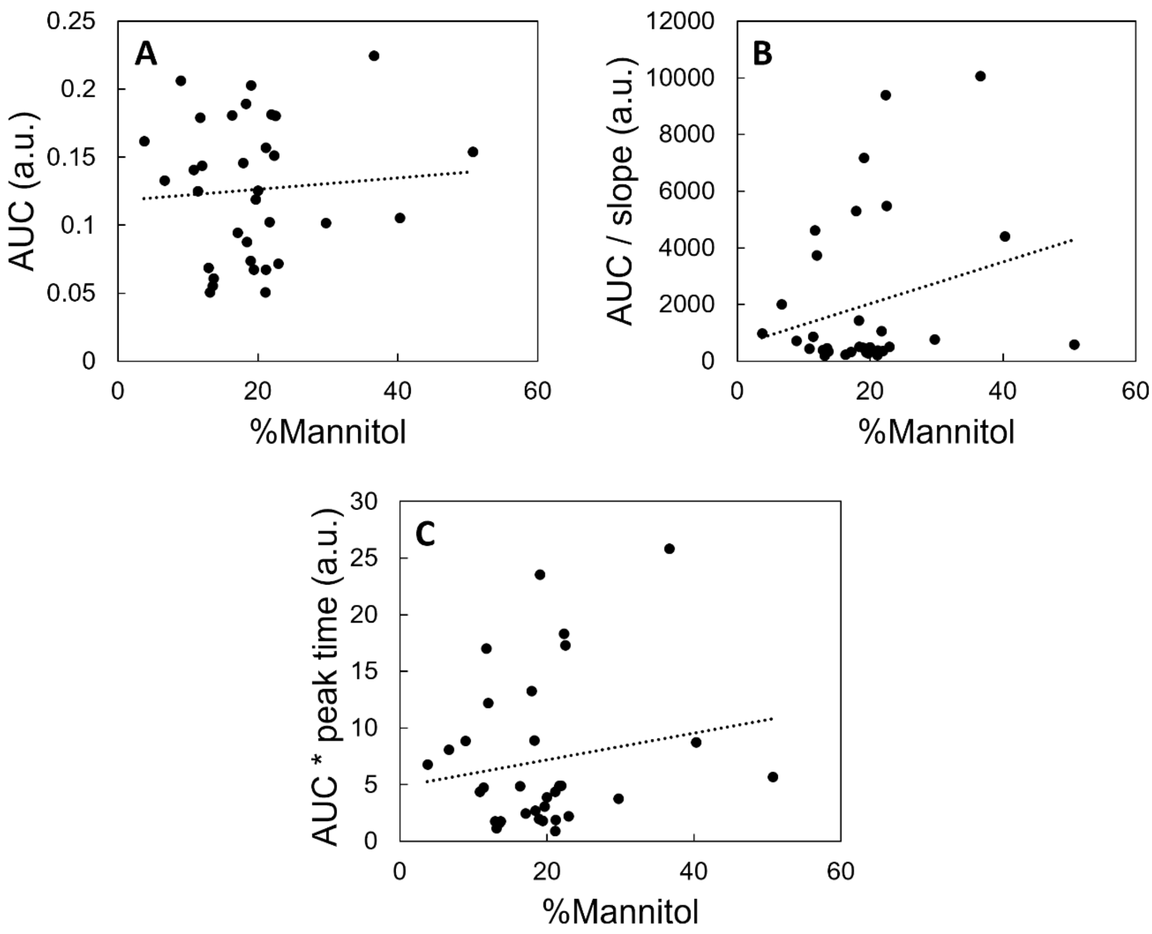

Figure S10. TFS vs. percentage mannitol (%M) correlation analysis – scatter plots. Graphs show TFS-derived AUC parameters (calculated at ultimate peak) against percentage mannitol recovery (%M). (**A**) AUC. (**B**) AUC / slope. (**C**) AUC * peak time. Dotted lines represent linear regression trend lines.

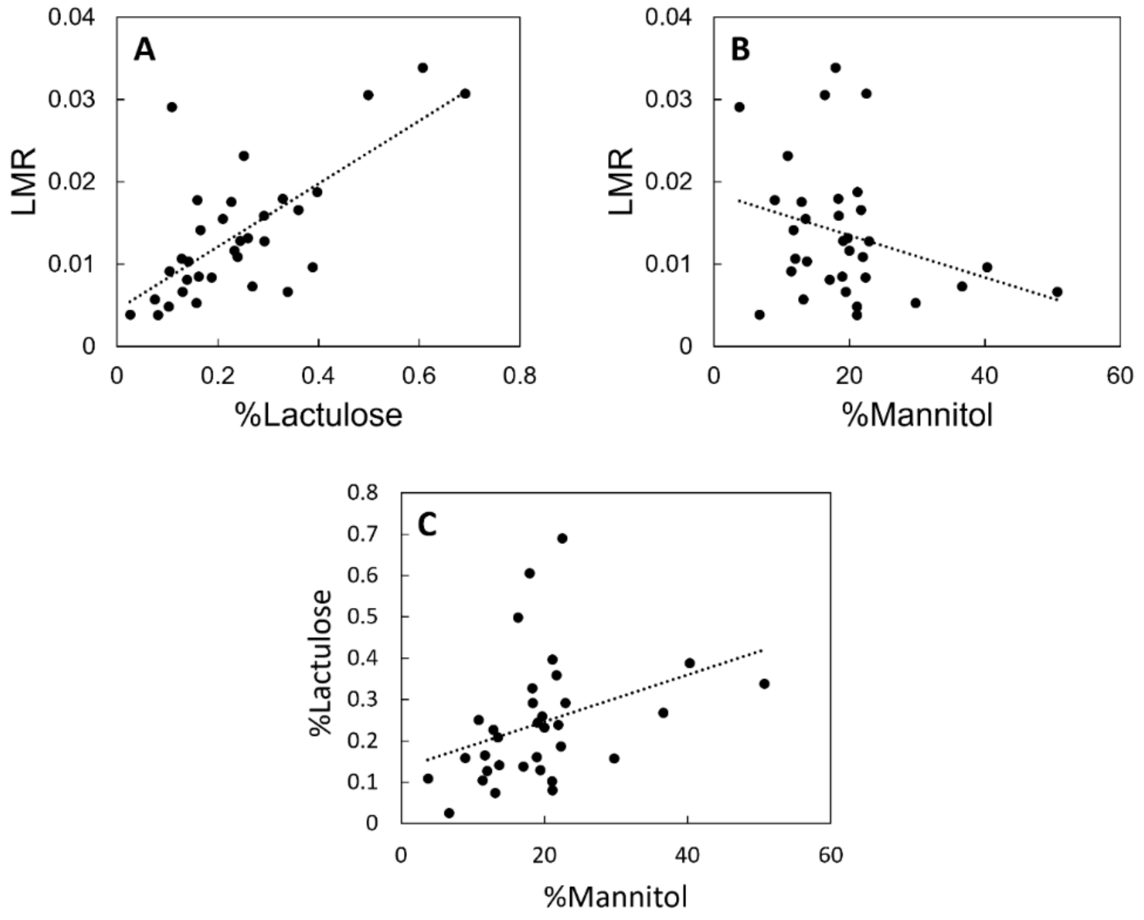

Figure S11. Analysis of correlations within L:M parameters. (**A**) Lactulose:mannitol ratio (LMR) vs. percentage lactulose recovery (%L). (**B**) LMR vs. percentage mannitol recovery (%M). (**C**) %L vs. %M. Dotted lines represent linear regression trend lines.

Supplementary Table

Table S1. TFS vs. percentage mannitol (%M) correlation analysis – statistical parameters. Spearman's rank correlation coefficients (r_s) and corresponding *p*-values for correlation of TFS-derived AUC parameters (AUC, AUC / slope, AUC * peak time) with percentage mannitol recovery (%M).

	**AUC**	**AUC / slope**	**AUC * peak time**
**%M**	r_s = -0.003<br>p = 0.988	r_s = 0.070<br>p = 0.692	r_s = 0.038<br>p = 0.831

Supplementary Methods

Measurement of lactulose and mannitol concentrations in urine samples using liquid chromatography – tandem mass spectrometry

To accurately measure lactulose and mannitol concentrations in urine samples, a targeted liquid chromatography – tandem mass spectrometry (LC-MS/MS) method was developed. The method was fully validated according to the established guidelines for bioanalytical method validation (see "M10 Bioanalytical Method Validation and Study Sample Analysis," available at https://www.fda.gov/media/162903/download).

Chemicals and materials

Lactulose and mannitol analytical standards were purchased from Sigma-Aldrich (Gillingham, UK). Stable isotope labelled internal standard of lactulose-$^{13}C_{12}$ was purchased from Merck Life Science (Gillingham, UK), and mannitol-$^{13}C_6$ was purchased from Molecular Dimensions Ltd (Rotherham, UK).

LC-MS grade acetonitrile was purchased from VWR International Ltd (Leicestershire, UK). LC-MS grade water, LC-MS grade methanol, and Ammonium acetate (CHROMASOLV™ LC-MS Ultra) were purchased from Fisher Scientific (Loughborough, UK).

LC-MS setup

The LC instrument setup consisted of a Waters ACQUITY UPLC solvent management system and a Waters 2777C external autosampler (Waters, Wilmslow, UK). Chromatographic separation was performed on a Waters ACQUITY UPLC BEH Amide 1.7 µm, 2.1 × 150 mm column (Waters Corporation) maintained at 60°C. Mobile phase A consisted of 5 mM ammonium acetate (pH 9.3) in water, and mobile phase B was 10% methanol in acetonitrile (v/v). The weak and the strong washes were 1:3 water/acetonitrile (v/v) and 100% isopropanol, respectively. Injection volume was 2 µl.

LC gradient was performed at 0.4 ml/min starting at 12% A (i.e. 12% mobile phase A, 88% mobile phase B) and maintained for 2 minutes followed by an increase to 30% A (70% B) at 4 minutes, which was maintained for the next 0.1 minutes. This was followed by an increase to 40% A (60% B) at 4.1 minutes and maintained until 6.7 minutes to elute all sample material from the column. At 7 minutes, LC gradient was returned to the initial conditions of 12% A (88% B) for re-equilibration, ending at 10 minutes.

MS detection was performed with a Waters Xevo TQ-S tandem quadrupole instrument (Waters, Wilmslow, UK) using electrospray ionization (ESI) in negative ion mode. Multiple reaction monitoring (MRM) was used for the quantification of lactulose and mannitol. The specific sugars and labelled standards MRM transitions are presented in Table S2. Nitrogen was used as desolvation gas, and argon was used as collision gas. The following source conditions were used for the MS run: capillary voltage of 2.5 kV; source offset of 80 V; desolvation temperature of 350°C; source temperature of 150°C, desolvation gas flow of 500 l/h; cone gas flow 150 l/h; nebulizer gas of 7.0 bar; collision gas of 0.1 ml/min.

Table S2. MS/MS transitions used for measuring lactulose, mannitol and their stable isotope labelled internal standards.

**Analyte**	**Parent *m/z***	**Quantifier *m/z***	**Qualifier *m/z***	**Collision Energy, eV**	**Cone Voltage, V**
Lactulose	**341.17**	**160.88**		6	22
	341.17		100.82	14	22
	341.17		178.82	8	22
Lactulose-$^{13}C_{12}$	**353.15**	**166.86**		6	20
	353.15		104.86	14	20
	353.15		184.87	6	20
Mannitol	**181.04**	**100.82**		16	14
	181.04		88.80	14	14
	181.04		58.84	22	14
Mannitol-$^{13}C_6$	**187.09**	**104.85**		14	4
			91.85	14	4
			73.79	18	4

Preparation of the calibration and quality control working solutions

Stock solutions of lactulose and mannitol and their stable isotope labelled internal standards of 1 mg/ml were prepared individually in LC-MS grade water and stored at -80°C. The stock solutions of lactulose and mannitol were mixed to obtain the calibration working solution (WS) for the highest concentrations of lactulose and mannitol – 1000 µg/ml and 10000 µg/ml for lactulose and mannitol respectively, which represented the upper limits of quantification (ULOQ). This WS was further diluted with LC-MS grade water to obtain a set of nine calibration WSs in the concentration range 20-1000 µg/ml for lactulose and 200-10000 µg/ml for mannitol.

Stable isotope labelled standards stock solutions were mixed and diluted with LC-MS grade water to obtain an internal standard mixture of 50 µg/ml each of lactulose-$^{13}C_{12}$ and mannitol-$^{13}C_6$.

WSs for quality control (QC) samples were prepared separately, diluting with LC-MS grade water and mixing the lactulose and mannitol stock solutions to obtain five concentration levels of both sugars that were within the linear range for each sugar but different from any calibration WS concentration levels (except for the lower limit of quantification, LLOQ). For lactulose and mannitol respectively, this resulted in QC WSs at concentrations of: 20 and 200 µg/ml (LLOQ QC); 100 and 1000 µg/ml; 250 and 2500 µg/ml; 500 and 5000 µg/ml; and 750 and 7500 µg/ml (high QC).

Sample preparation for LC-MS/MS analysis

Study urine samples stored at -80°C were thawed overnight at 4°C and then vortex-mixed. Aliquots of 80 µl of each sample were added to 96-deep-well polypropylene plates (2 ml, Eppendorf) and were dried under a gentle stream of nitrogen at room temperature. Subsequently, the dried samples were

reconstituted in 20 µl of LC-MS grade water. Samples were vortex mixed, and 20 µl of the stable isotope labelled internal standards solution (containing 50 µg/ml each of lactulose-$^{13}C_{12}$ and mannitol-$^{13}C_6$ in LC-MS water) was added to each reconstituted sample. The samples were vortex mixed for two minutes before adding 120 µl of cold 1:2 methanol:acetonitrile mixture. The plates were sealed and mixed at 1400 rpm for two minutes at 4°C (MixMate, Eppendorf). The plates were then centrifuged for ten minutes at 3486g and 4°C prior to the LC-MS/MS analysis.

Preparation of the calibration solutions and QC samples followed a similar protocol, starting with aliquots of 20 µl of each concentration level WS of lactulose and mannitol mixture in LC-MS water, followed by addition of 20 µl of the stable isotope labelled internal standards solution (containing 50 µg/ml each of lactulose-$^{13}C_{12}$ and mannitol-$^{13}C_6$) and 120 µl of cold 1:2 methanol:acetonitrile mixture.

A double blank (DB) solution was prepared as method diluent containing 1:1:2 LC-MS grade water:methanol:acetonitrile. A single blank (SB) solution was prepared as described above for calibration solutions but using LC-MS grade water instead of the calibration WS.

Sample formatting and run order of LC-MS/MS analysis

Urine samples were analysed as randomised blocks of samples collected from each patient at baseline and after sugar intake. Each analytical plate comprised 40 study samples, a set of nine calibration solutions with SB solution preceding it, and three full sets of QC samples.

The analytical run was initiated by a SB injection prior to the calibration set and one full QC series. The second QC series was injected every eight study samples, and the third QC set was analysed at the end of the study samples analysis. DB was injected after each ULOQ solution and high QC sample to avoid any carryover in subsequent samples.
